# Lactobacillus paracasei CNCM I-3689 reduces vancomycin-resistant Enterococcus persistence and promotes Bacteroidetes resilience in the gut following antibiotic challenge

**DOI:** 10.1038/s41598-018-23437-9

**Published:** 2018-03-23

**Authors:** Laureen Crouzet, Muriel Derrien, Claire Cherbuy, Sandra Plancade, Mélanie Foulon, Benjamin Chalin, Johan E. T. van Hylckama Vlieg, Gianfranco Grompone, Lionel Rigottier-Gois, Pascale Serror

**Affiliations:** 1 Micalis, INRA, AgroParisTech, Université Paris-Saclay, 78350 Jouy-en-Josas, France; 2 Danone Nutricia Research, F-91120 Palaiseau, France; 3 Maiage, INRA, Université Paris-Saclay, 78350 Jouy-en-Josas, France; 4 Present Address: Medis, INRA Clermont-Ferrand-Theix, 63122 Saint-Genès-Champanelle, France; 5 Present Address: Chr. Hansen AS, Hoersholm, Denmark; 6 Present Address: Instituto Nacional de Investigación Agropecuaria, Montevideo, Uruguay

## Abstract

Enterococci, in particular vancomycin-resistant enterococci (VRE), are a leading cause of hospital-acquired infections. Promoting intestinal resistance against enterococci could reduce the risk of VRE infections. We investigated the effects of two *Lactobacillus* strains to prevent intestinal VRE. We used an intestinal colonisation mouse model based on an antibiotic-induced microbiota dysbiosis to mimic enterococci overgrowth and VRE persistence. Each *Lactobacillus* spp. was administered daily to mice starting one week before antibiotic treatment until two weeks after antibiotic and VRE inoculation. Of the two strains, *Lactobacillus paracasei* CNCM I-3689 decreased significantly VRE numbers in the feces demonstrating an improvement of the reduction of VRE. Longitudinal microbiota analysis showed that supplementation with *L*. *paracasei* CNCM I-3689 was associated with a better recovery of members of the phylum Bacteroidetes. Bile salt analysis and expression analysis of selected host genes revealed increased level of lithocholate and of ileal expression of *camp* (human LL-37) upon *L*. *paracasei* CNCM I-3689 supplementation. Although a direct effect of *L*. *paracasei* CNCM I-3689 on the VRE reduction was not ruled out, our data provide clues to possible anti-VRE mechanisms supporting an indirect anti-VRE effect through the gut microbiota. This work sustains non-antibiotic strategies against opportunistic enterococci after antibiotic-induced dysbiosis.

## Introduction

The human gastrointestinal (GI) tract is colonised by a dense and diverse microbial community referred to as the gut microbiota. This highly complex microbial ecosystem is involved in many host physiological processes including improvement of the intestinal epithelial barrier, education of the immune system, and nutrient acquisition^1^. In adult mammals, the gut microbiota is dominated by two bacterial phyla—the Firmicutes and the Bacteroidetes—but the bacterial species present are highly diverse^2,3^. Mounting evidence demonstrates that some members of the sub-dominant fraction of the gut microbiota, referred to as pathobionts, can harbor potential pathogenic features. Pathobionts can expand under some circumstances resulting in the disruption of a well-balanced microbial ecosystem and suspected to be involved in opportunistic infections or chronic diseases^4,5^. In particular, pathobiont proliferation represents a threat in immunocompromised and frail elderly people causing infectious diseases^6–9^.

Enterococci are natural inhabitants of the subdominant human intestinal microbiota in adults^2,3^. They are considered pathobionts as many enterococci are harmless for healthy humans, but can be pathogenic under certain circumstances (e. g. prolonged antibiotic treatments, severe underlying diseases, and impaired immune system); causing urinary tract and intra-abdominal infections, bacteremia and infective endocarditis. Furthermore, *Enterococcus* spp. contribute to community-acquired intra-abdominal infections^10,11^ and count among the ten most frequently isolated micro-organisms in healthcare-associated infections^12–14^. As enterococci are intrinsically resistant to many antibiotics such as penicillin and cephalosporins, broad-spectrum antibiotic treatment is one of the conditions of overgrowth of enterococci at the expense of major members of the gut microbiota. Intestinal expansion of *Enterococcus* spp. is associated with an increased risk of developing bloodstream infection with the same bacterial species^15,16^. *E*. *faecalis* accounts for more than 60% of the hospital-acquired enterococcal infections^17–19^. Given the importance of gastrointestinal colonisation and proliferation as primary steps of *E*. *faecalis* infectious process and transmission between patients, prevention of *E*. *faecalis* overgrowth and persistence in the GI tract appears a good approach to limit the risks of infection following antibiotic treatment.

It has been previously demonstrated that ingested bacteria or probiotics can increase resistance mechanisms against intestinal pathogens^20,21^. This beneficial effect of probiotic strains can involve direct inhibitory effect by competition for nutrients or killing of the pathogen by an inhibitory molecule or indirect effect such as a positive impact on the gut microbiota composition or on the host defense mechanisms^22^. Despite recent clinical success of fecal microbiota transplantation in the reduction of VRE and identification of mouse commensal strains that restore colonisation resistance against VRE, relatively few studies evaluating the use of *Lactobacillus* spp. strains to prevent or limit VRE colonisation and overgrowth have been reported^23–25^. For instance, two studies reported a beneficial effect of *Lactobacillus rhamnosus* GG in human^26,27^. The use of strains with a qualified presumption of safety appears less problematic than fecal microbiota transfer^25,28,29^. As members of the lactic acid bacteria studied for health-promoting effects, several strains of lactobacilli were shown to reinforce the epithelial intestinal barrier and prevent development of pathogens^30^. Among them, *Lactobacillus paracasei* CNCM I-3689 decreases translocation and dissemination of *Listeria monocytogenes* in gnotobiotic and conventional mice^31^ and *L*. *rhamnosus* CNCM I-3690 improves intestinal barrier integrity in a murine model and exerts anti-inflammatory properties^32,33^.

We previously adapted an *E*. *faecalis* colonisation model in mice with conventional microbiota as developed by Donskey *et al*.^34,35^. In this model, mice are pre-treated with clindamycin that causes an imbalance of the gut microbiota including an increase of endogenous enterococci and allows transient colonisation of *E*. *faecalis* V583 strain, a representative of the leading hospital adapted lineage of *E*. *faecalis* in the United States and in several European countries^36,37^. The aim of this work was to evaluate the effect of the strains *L*. *paracasei* CNCM I-3689 and *L*. *rhamnosus* CNCM I-3690 on the colonisation and persistence of the *E*. *faecalis* V583 strain. We measured transient colonisation and persistence of VRE in the intestine of mice upon supplementation with *Lactobacillus* spp. strains. We observed an improvement of VRE reduction by *L*. *paracasei* CNCM I-3689. The effect of this strain on the gut microbiota and on the expression of a selection of host genes was analysed leading us to propose that part of the *L*. *paracasei* CNCM I-3689 anti-VRE effect may rely on a faster recovery of members of the phylum Bacteroidetes.

## Results

### Lactobacillus paracasei CNCM I-3689 reduces E. faecalis V583 persistence level

**Figure 1:**
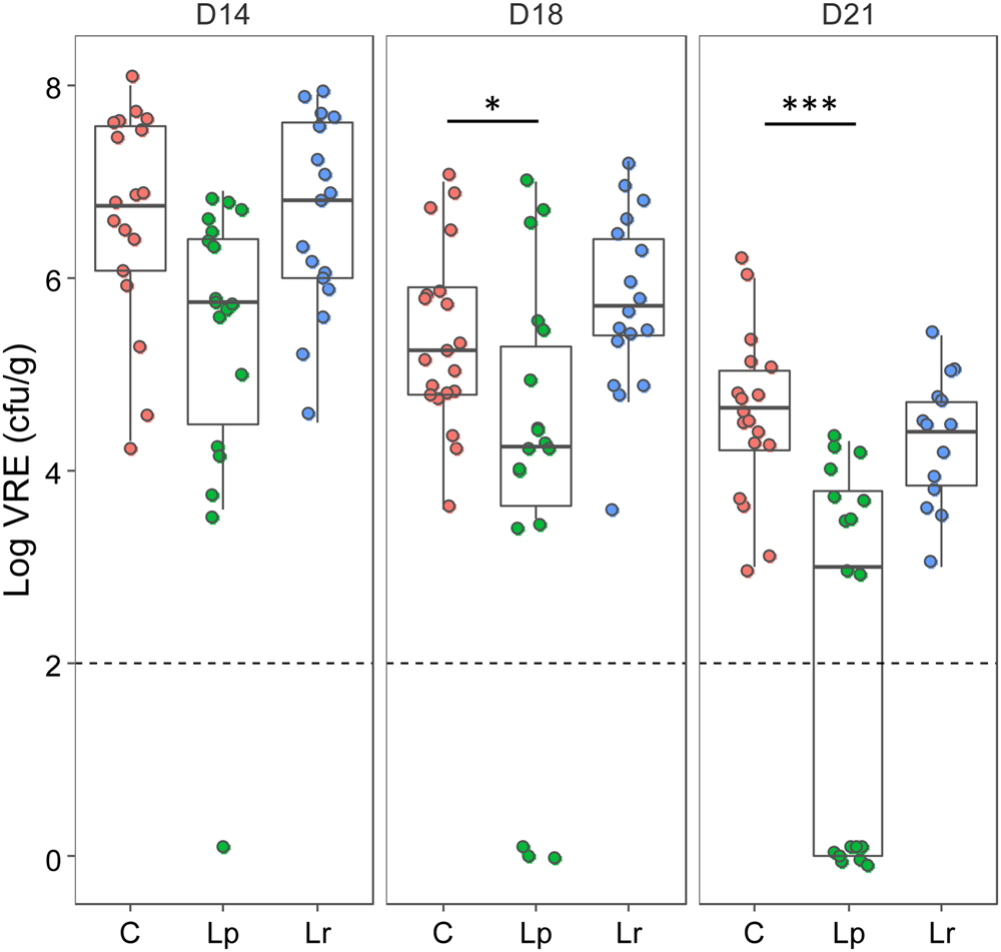
*L*. *paracasei* CNCM I‐3689 reduces the fecal levels of VRE *E*. *faecalis* V583 in the intestinal tract. *E*. *faecalis* V583 counts (CFU/g) at D14, D18 and D21 in mice receiving strain *L*. *paracasei* CNCM I-3689 (Lp), *L*. *rhamnosus* CNCM I-3690 (Lr) or NaCl (C). Each dot represents one mouse (n = 13 to 18). Horizontal bars represent the median for each condition and the dashed line indicates the detection limit. Statistical tests were performed using a Mann–Whitney test. Asterisks indicate a p-value considered statistically significant (*P = 0.057; ***P = 0.001).

**Figure 2:**
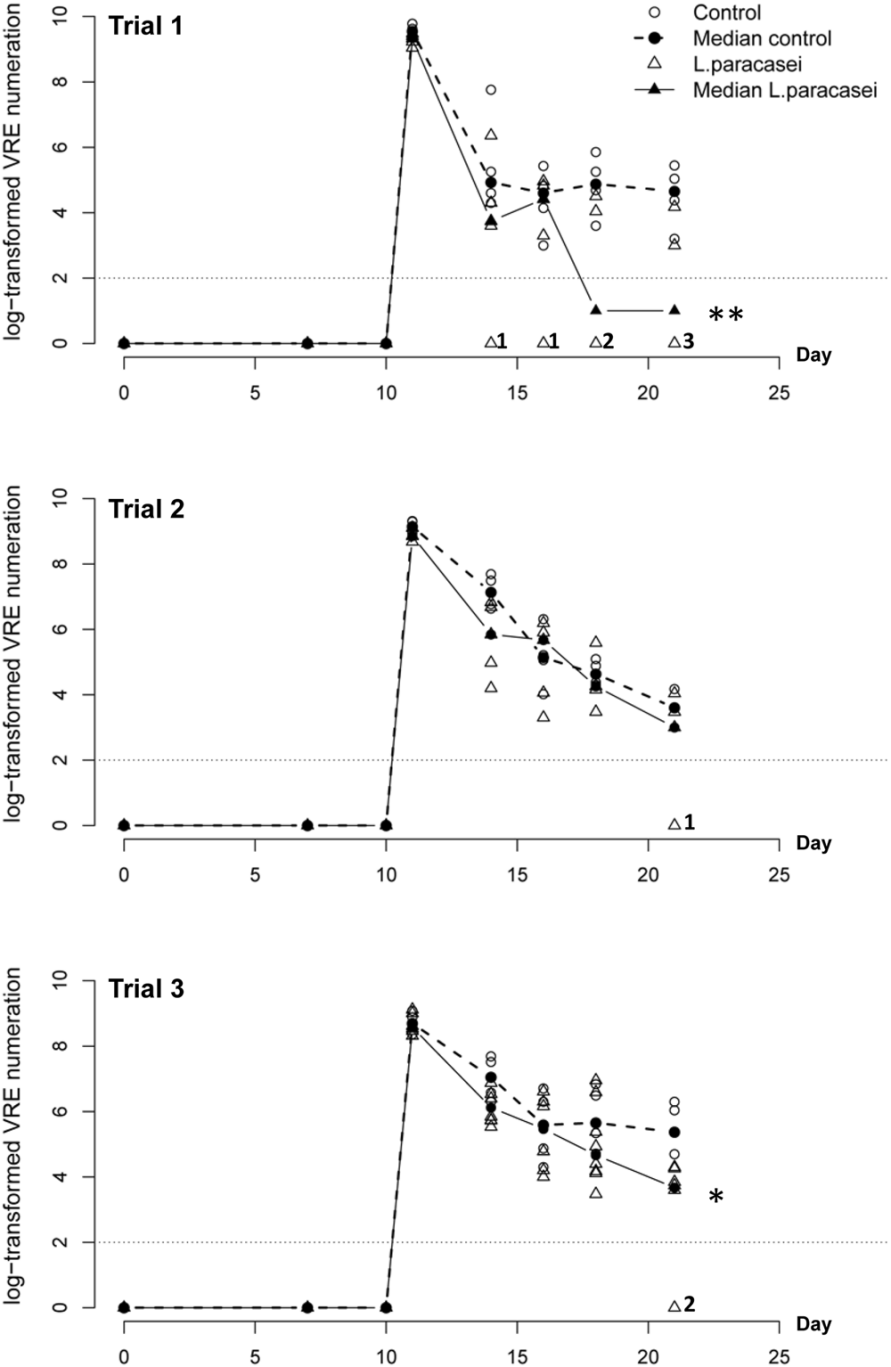
Kinetics of fecal levels of VRE in trials 1, 2 and 3. Trial 1 (n = 5 mice per group), trial 2 (n = 4 mice for control and n = 5 for *L*. *paracasei* group), trial 3 (n = 5 mice for control group and n = 8 for *L*. *paracasei* group). Values below the detection level at 10^2^ were set to zero and numbers on the right of the empty triangles indicate the number of mice with VRE below the detection level. The continuous and dashed line curves show the median of log-transformed numeration from mice in control and *L*. *paracasei* groups computed at each time point. For time points with more than half of the values below 10^2^ the mean of the two extreme values is represented, as the median was not uniquely defined and laid between 0 and the smallest non-zero value. Asterisks indicate a P-value considered statistically significant (*P < 0.05; **P < 0.01).

We previously showed that transient intestinal colonisation of clindamycin-treated mice by the vancomycin-resistant *E*. *faecalis* strain V583 paralleled the overgrowth of endogenous enterococci^35,38^. We used this model to examine the effect of the strains *L*. *paracasei* CNCM I-3689 and *L*. *rhamnosus* CNCM I-3690 on the colonisation and persistence of *E*. *faecalis* V583 according to the experimental protocol depicted in Supplementary Figure S1. After an adaptation period (D0), mice received a daily dose of 10^9^ CFU of strain *L*. *paracasei* CNCM I-3689, *L*. *rhamnosus* CNCM I-3690 or control solution for the duration of the experiment (D21). After the first week of supplementation (D7), animals received clindamycin for 3 days (D7 to D9) and were then inoculated with strain *E*. *faecalis* V583 at D10. Numbers of strain *E*. *faecalis* V583, total enterococci, and lactobacilli were monitored. Three independent trials (trial 1, 2 and 3, Supplementary Figure S1) were carried out. In this model, the inoculated strain *E*. *faecalis* V583 transiently colonised the GI tract, reaching a maximum of 5 × 10^8^ to 5 × 10^9^ CFU/g, one day (D11) after inoculation in the control, *L*. *paracasei* CNCM I-3689 and *L*. *rhamnosus* CNCM I-3690 groups. In all trials the fecal counts of *E*. *faecalis* V583 decreased and stabilised at 10^5^ CFU/g nine days (D18) after the end of antibiotic treatment in the control and *L*. *rhamnosus* groups. Conversely we observed a higher number of mice with *E*. *faecalis* V583 under the detection limit from D14 to D21 indicating a progressive reduction of carriage of *E*. *faecalis* V583 in the group receiving *L*. *paracasei* CNCM I-3689 (Fig. 1). At D21 *E*. *faecalis* V583 number was below the detection level (<10^2^ CFU/g) in eight out of eighteen of the mice receiving *L*. *paracasei* CNCM I-3689. Comparison of the kinetics of VRE for *L*. *paracasei* and control groups in the different experiments (Fig. 2) established that numbers of *E*. *faecalis* V583 were significantly reduced in trials 1 (P = 0.0079) and 3 (P = 0.013). In contrast, strain *L*. *paracasei* CNCM I-3689 did not show significant reduction on numbers of strain *E*. *faecalis* V583 in trial 2. This result may be linked to a faster wash-out of *E*. *faecalis* V583 in the control group of this trial, indicative of a lower persistence of V583. Together these data showed that strain *L*. *paracasei* CNCM I-3689 contributes to intestinal reduction or clearance of vancomycin-resistant *E*. *faecalis* V583.

Clindamycin treatment was concomitant with transient increase of total enterococci, which reached the highest level (~5 × 10^9^CFU/g) two days after the end of the antibiotic treatment (D11) and decreased to an average level (~10^7^ cfu/g) within the next 5 days (Figure S2). Lactobacilli population also reached a maximum (>10^10^ CFU/g) at D11 (Figure S2). Similarly to total enterococci, no difference on total lactobacilli was detected between the three animal groups, indicating that administration of *L*. *paracasei* CNCM I-3689 or *L*. *rhamnosus* CNCM I-3690 had no major effect on total enterococci and lactobacilli when assessed using cultivation based approach.

### Strain *L*. *paracasei* CNCM I-3689 improves resilience of Bacteroidetes members after clindamycin treatment

To examine whether strain *L*. *paracasei* CNCM I-3689 had an effect on the intestinal microbiota and to characterise changes in the microbiota that could be associated with a reduction of *E*. *faecalis* V583, we analysed the fecal microbiota of control and *L*. *paracasei* CNCM I-3689-supplemented mice by 16S rRNA gene sequencing. To circumvent potential interference of the presence of VRE on the microbiota, we performed two trials (Trials 4 and 5) by omitting inoculation of *E*. *faecalis* V583 at D11 as depicted in Supplementary Figure S1. Microbiota diversity and composition were compared for 3 to 8 mice for trials 1, 2, 4 and 5 at baseline (D0), 1 week after *L*. *paracasei* CNCM I-3689 supplementation (D7), and one (D11) and eleven (D21) days after the cessation of the clindamycin-treatment. In addition, microbiota was analysed the day before *E*. *faecalis* V583 inoculation (D10) for trials 1 and 2, and four days after the end of the clindamycin-treatment (D14) for trials 4 and 5. For trial 3, microbiota was analysed at baseline (D0) and 11 days after inoculation of *E*. *faecalis* V583 (D21) only. A total of 183 samples were analysed (Supplementary Table S1).

**Figure 3:**
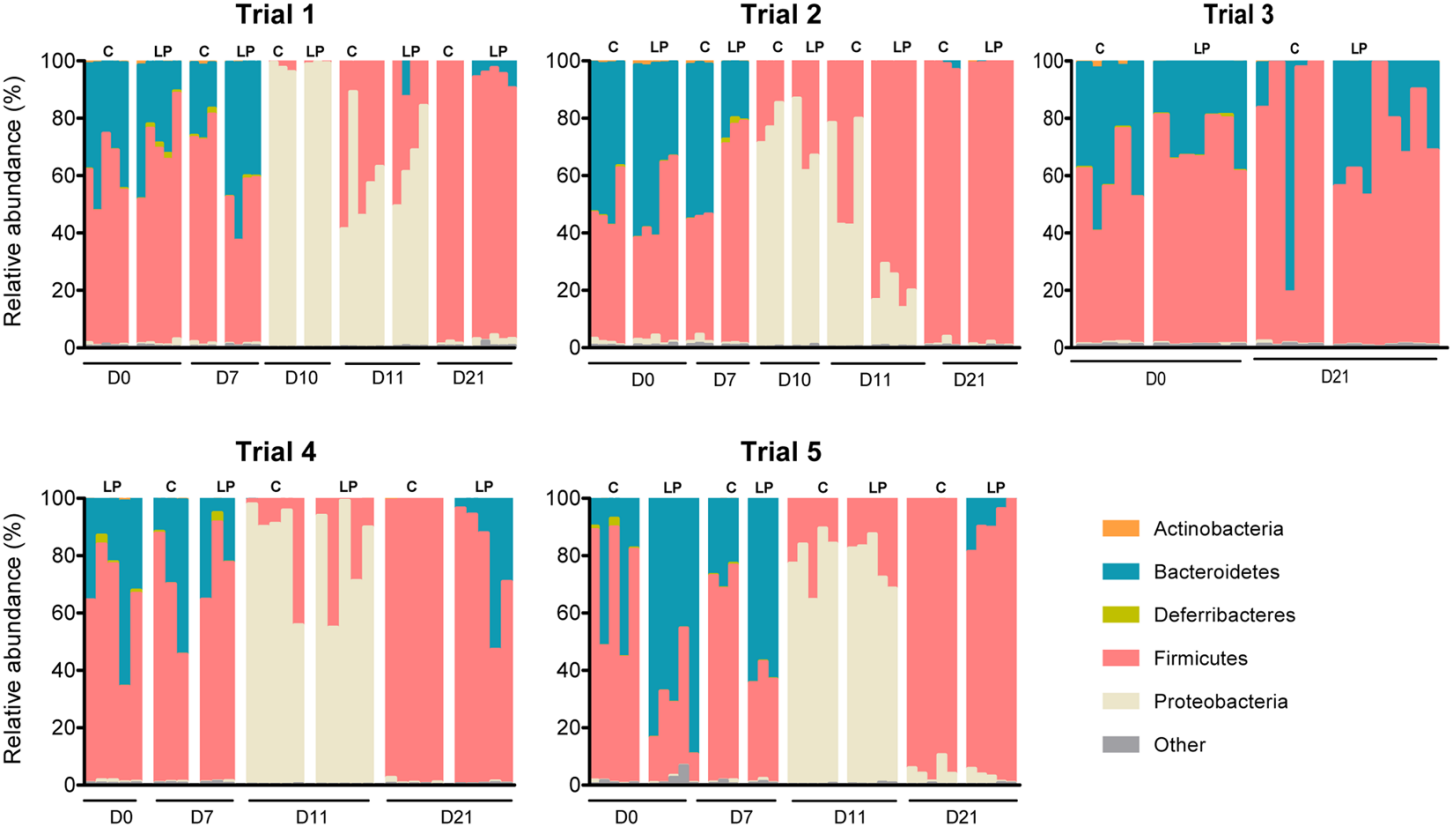
*L*. *paracasei* CNCM I‐3689 supplementation improves Bacteroidetes recovery. Kinetics of the microbiota at phylum level at all days for control and *L*. *paracasei* supplemented groups in each trial. Trials 1 to 3 were performed in presence of *E*. *faecalis* V583 and trials 4 and 5 in absence of *E*. *faecalis* V583.

Because bacterial communities varied between mice and trials at baseline at genus level, phylum-based analysis is presented for each trial (Fig. 3 and Supplementary Table S2). At baseline, the mouse gut microbiota was dominated by Firmicutes and Bacteroidetes, accounting for >95% of total microbiota with presence of Proteobacteria and Actinobacteria at lower levels (accounting for less than 2%). Using a permutational analysis of variance (PERMANOVA), no major effect of *L*. *paracasei* CNCM I-3689 on microbiota composition was detected after daily supplementation for seven days (Supplementary Table S3). The effect of clindamycin treatment (D10 and D11) on gut microbiota was evaluated on both alpha and beta-diversity. A significant reduction of richness (as measured by number of Operational Taxonomic Units (OTUs)) and evenness (as measured by Shannon index) of the gut microbiota was observed (Supplementary Figure S3, P < 0.05). A significant shift in microbiota represented by a principal coordinate analysis (Supplementary Figure S4) was observed in each group of mice following clindamycin treatment (D11) for both weighted and unweighted UniFrac distances (PERMANOVA tests P = 0.001). At taxonomical level, the phylum Bacteroidetes was severely reduced to non detectable level, while a drastic bloom of Proteobacteria was observed (increase from less than 2% to up to 97%) following clindamycin administration. Eleven days (D21) after the end of the clindamycin treatment, despite a decrease of Proteobacteria close to baseline level (~2%) and an increase of the Firmicutes, the microbiota composition was still different from baseline, regardless of *L*. *paracasei* supplementation and *E*. *faecalis* V583 inoculation (Supplementary Table S4). In trials 4 and 5, Bacteroidetes were detected in all animals receiving *L*. *paracasei* CNCM I-3689 and absent in the control group (P = 10^−5^). A lower abundance of Firmicutes (P = 0.0039) and Proteobacteria (P = 0.075) following *L*. *paracasei* CNCM I-3689 supplementation was also observed. These results support that administration of *L*. *paracasei* CNCM I-3689 improved microbiota recovery after cessation of antibiotic-treatment. While all animals receiving *L*. *paracasei* CNCM I-3689 had detectable level of the *Bacteroides* genus, none had in the control group. Other genera from Bacteroidetes were affiliated mostly to the Bacteroidales order. They included an unknown genus from the S24-7 family and *Parabacteroides* (*Porphyromonadaceae*) in 70 and 60% of the mice, respectively (Supplementary Figure S5). When mice were inoculated with VRE, the phylum Bacteroidetes was more abundant upon *L*. *paracasei* CNCM I-3689-supplementation in trials 1 (with the exception of an outlier) and 3, but not in trial 2 (Fig. 3). The effect of *L*. *paracasei* CNCM I-3689 on the increase of Bacteroidetes (P = 0.0445) was statistically confirmed when data of trials 1 and 3 were pooled, supporting that Bacteroidetes recovery is associated with improved VRE reduction. Notably, the improved microbiota recovery in the absence of *E*. *faecalis* V583 suggests that *E*. *faecalis* V583 interferes on the effect of *L*. *paracasei* CNCM I-3689 supplementation. Furthermore, to examine whether a *Lactobacillus* strain with no anti-VRE effect promoted Bacteroidetes recovery, we performed a trial with *L*. *rhamnosus* CNCM I-3690 supplementation and without VRE inoculation. We analysed by 16S sequencing the fecal microbiota of control and *L*. *rhamnosus* CNCM I-3690 -supplemented mice collected at D0 and D21. Microbiota composition analysis at the phylum level revealed no difference between control and supplemented groups at D21 (Figure S6 and Supplementary Table S5). Notably, Bacteroidetes was not detected in any of the groups at D21. Together, our results show that of the two strains tested *L*. *paracasei* CNCM I-3689 specifically improves the microbiota recovery after antibiotic induced dysbiosis by promoting resilience of some genera from Bacteroidetes, especially *Bacteroides* and other genera of the *Bacteroidales* order. However, *L*. *paracasei* CNCM I-3689 has no major effect on the microbiota in non-antibiotic treated mice, neither during antibiotic treatment.

### *L*. *paracasei* CNCM I-3689 does not inhibit growth of *E*. *faecalis* V583 *in vitro*

Our *in vivo* studies have demonstrated an impact of *L*. *paracasei* CNCM I-3689 strain on *E*. *faecalis* persistence in feces. To gain insights on the underlying mechanisms we investigated whether *L*. *paracasei* CNCM I-3689 strain exerts a direct effect on the growth of *E*. *faecalis* V583 *in vitro* through the production of a diffusible inhibitory molecule. We cultured *E*. *faecalis* V583 in the presence of culture supernatant of *L*. *paracasei* CNCM I-3689 neutralised at pH 7.0. No effect of *L*. *paracasei* CNCM I-3689 supernatant was observed on growth of *E*. *faecalis* V583 under the experimental conditions used (Supplementary Figure S7).

### *L*. *paracasei* CNCM I-3689 modulates intestinal host responses in parallel to the reduction of fecal levels of VRE

**Figure 4:**
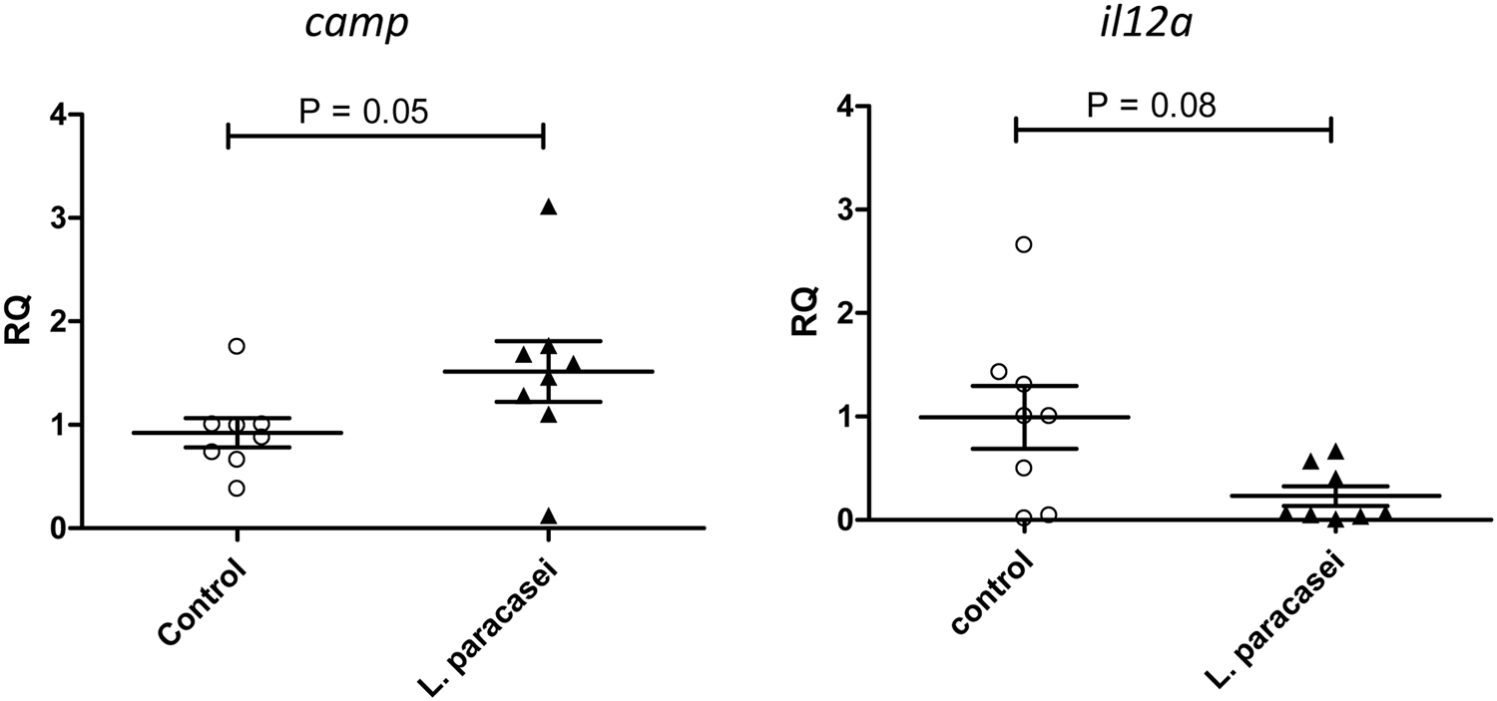
*L*. *paracasei* CNCM I‐3689 supplementation modulates the expression of *camp* and *il12a* expression in the ileum. Ileal *camp* and *il12a* gene expression were analysed by QPCR in Control or *L*. *paracasei* groups. RQ is the relative abundance of *camp* or *Il12a* mRNA normalised to those of *gapdh* and compared with values for control mice. 8 mice were analysed in both groups. Each point on the graph corresponds to an individual. *Significantly different from control mice values (P = 0.05).

We further investigated whether the impact of *L*. *paracasei* CNCM I-3689 on the reduction of *E*. *faecalis* V583 was associated with modulation of the intestinal host responses in ileum, a major site of immune response. Expression of 42 genes involved in various intestinal defenses mechanisms such as cytokines, immune responses, regulation of cell proliferation and differentiation, the intestinal barrier, antimicrobial peptides was analysed using a custom-designed TaqMan Low Density Array (Supplementary Table S6). Analysis was carried out on the ileum of eight mice for control and *L*. *paracasei* groups of trials 1 and 3 at D21 where maximum effect of VRE reduction by *L*. *paracasei* CNCM I-3689 was observed. We identified two genes of interest when differences between control and *L*. *paracasei* groups were ranked by p-value: *camp* that encodes the anti-microbial peptide cathelicidin, and *il12a* that encodes the p35 subunit of the pro-inflammatory cytokine IL-12 (Supplementary Table S3). We further analysed the expression of these genes by single Taqman assays (Fig. 4). Expression of *camp* was increased in *L*. *paracasei* compared to control group (P = 0.05). We also observed a trend of decreased expression of *il12a* in *L*. *paracasei* compared to control group (P = 0.08). We also investigated the effect of *L*. *paracasei* administration on ileal and colonic epithelium structure through the analysis of tight junction and cell adhesion proteins ZO-1, claudin-1 and claudin-2 and of proliferation markers Ki67 and PCNA. We found no differences for ZO-1 and claudins at the ileal and colonic levels (data not shown). In contrast, our data revealed a significant increase both of Ki67- and PCNA-positive cells in the colonic epithelium after *L*. *paracasei* CNCM I-3689 supplementation (Supplementary Figure S8), suggesting *L*. *paracasei* CNCM I-3689 could contribute to the dynamic of the regeneration process of the small intestine.

### Bile acids and SCFA analysis

We further investigated the impact of *L*. *paracasei* CNCM I-3689 on metabolites that were previously reported to have anti-pathogenic effects. SCFA were quantified in cecal contents at D21 from trials 1, 4 and 5, in which Bacteroidetes was increased following *L.* *paracasei* CNCM I3689 supplementation. Regarding, SCFA, there was no difference between controls and *L*. *paracasei* treated-mice in absolute amounts for acetate and butyrate (Supplementary Table S7). However *L*. *paracasei* CNCM I-3689 increased the absolute amount of propionate compared to control group (P < 0.05 in trials 1 and 5). Noticeably, very low amount of propionate detected in control groups may emphasise the difference.

In addition, bile acids were analysed from cecal contents at D21 from trial 3. Six primary and secondary bile acids were found to dominate the cecal content of control and *L*. *paracasei* mice: cholic and taurocholic acids (primary), and deoxycholic, lithocholic, ursodeoxycholic, and taurodeoxycholic acids (secondary). Although the variability was found to be high within groups, there was a trend towards higher level of the secondary bile acid lithocholic acid in *L*. *paracasei* treated mice (P = 0.057) (Supplementary Figure S9).

## Discussion

Among intestinal pathobionts, vancomycin-resistant enterococci (VRE) are a leading cause of health-care associated and community-acquired infections associated with increased morbidity of patients and hospital costs. Promotion of the colonisation resistance provided by the gut microbiota is an attractive non-antibiotic alternative to minimise the colonisation and persistence of VRE. Few clinical studies have established that probiotics enhance intestinal barrier against intestinal pathogens^20,21,39^. The development of probiotics against multi-antibiotic resistant nosocomial enterococci is a significant area of unmet medical need with the goal of reducing pathogen transmission and dissemination. This study highlights that strain *L*. *paracasei* CNCM I-3689 reduces the fecal level of VRE and promotes the recovery of some dominant members of the gut microbiota, mostly Bacteroidetes, after antibiotic-induced dysbiosis. We found that Camp (LL-37) and IL-12a are candidate host factors modulated by the supplementation with *L*. *paracasei* CNCM I-3689 strain in the presence of *E*. *faecalis*. The candidate host factors are acting mainly at two different levels in terms of immune response: Camp as an antimicrobial peptide via innate mucosal immunity and IL-12a as a potential link with adaptive immunity via gut macrophages and DCs that could be modulated upon *L*. *paracasei* CNCM I-3689 supplementation. Although the expression of inhibitory molecules may differ *in vivo* and *in vitro*, the absence of *in vitro* inhibition of the strain *L*. *paracasei* CNCM I-3689 against V583 is not in favor of a direct anti-VRE mechanism such as bacteriocins as observed by Millete *et al*.^40^ or recently by Kommineni *et al*.^41^. Even if other direct mechanisms for the *L*. *paracasei* CNCM I-3689-mediated anti-VRE effect such as nutrient competition may occur, one possible mechanism could be indirect and involve Bacteroidetes and/or the host response.

Given complex interactions among microorganisms in the human microbiota, preventing carriage of antimicrobial-resistant enterococci may be challenging while decreasing the load may be sufficient to prevent infection and dissemination. We assessed the anti-VRE potential of two *Lactobacillus* strains showing interindividual differences combined with several independent trials. In this worst-case scenario, no preventive effect on the VRE overgrowth of the *Lactobacillus* strains supplementation was observed. Impact of probiotics on composition of human fecal microbiota in healthy adults has been reported to be minor^42^. Because of interindividual variability, larger scale trials and greater probing depth are needed to assess if these *Lactobacillus* strains have any effect on eubiotic microbiota. Alternatively, the response of disrupted microbiota to probiotics appears relevant^43^. Yet, we repeatedly detected a significant reduction of persisting VRE upon supplementation with *L*. *paracasei* CNCM I-3689 in trials 1 and 3, although not all mice responded. Interindividual variation of baseline microbiota has been shown to be a factor that participates to the response to a given intervention, either dietary, pharmaceutical or fecal transplantation^42,44^. Another confounding factor in our experimental conditions is that coprophagy of feces with high amount of VRE can fuel cross-contaminations by VRE. However, our results suggest that supplementation with *L*. *paracasei* CNCM I-3689 rather improves reduction of persisting *E*. *faecalis* V583 than eradicates *E*. *faecalis* from the gut microbiota. No effect of the strain *L*. *rhamnosus* CNCM I-3690 was observed although efficacy of *Lactobacillus* spp. strains from the same species against VRE was reported. The strain *L*. *rhamnosus* GG reduced at least transiently the number of VRE in adult or children VRE-positive patients within four and three weeks^26,27^, whereas supplementation of adult VRE-positive patients with *L*. *rhamnosus* Lcr35r had no effect on VRE carriage^44^. However, Vidal *et al*. reported that this strain was able to decrease VRE *Enterococcus faecium* in mice up to 34 days after inoculation. These studies highlight that probiotic effects are strain specific. Assessment of anti-VRE effect at the preclinical level in animal trials remains a way to increase the chance to identify good candidates against VRE in human.

Resilience of microbiota following an external challenge, including antibiotics is of high clinical relevance as a less resilient microbiota might predispose to diseases^45^. Administration of clindamycin has long lasting effect on members of Bacteroidetes phylum in human and mice, mainly *Bacteroidaceae*^46–49^. Here we showed that supplementation with *L*. *paracasei* CNCM I-3689 is correlated with partial recovery of the gut microbiota for instance Bacteroidetes phylum, mostly Bacteroidales order and with a higher reduction in VRE levels. Interestingly, significant enrichment of obligate anaerobes including Bacteroidetes has been inversely associated with *E*. *faecalis* levels^50^. Reintroduction of Bacteroidetes to antibiotic-treated mice would allow to assess if Bacteroidetes recovery and reduction in levels of fecal VRE are corelated or independent events. Ingestion of bacterial strains that can hasten the recovery of members of Bacteroidales order is therefore of high relevance as they include dominant members of the gut microbiota, in mice and human^51^ that have important roles on physiological and immunological functions^52^. Identification of the species and the underlying mechanisms that may mediate *L*. *paracasei* CNCM I-3689 effect on VRE reduction is challenging, especially because the presence of VRE seems to interfere with recovery of Bacteroidetes. Ubeda *et al*.^53^ demonstrated that reintroduction of a diverse intestinal microbiota containing *Barnesiella intestihominis*, a member of the Bacteroidales order, to densely VRE-colonised mice eliminates VRE from the intestinal tract. Of note, prospective analysis of patients’ gut microbiota undergoing allogeneic hematopoietic stem cell transplantation substantiated that high level of *Barnesiella* spp. correlates with resistance to intestinal VRE domination^53^. Recently, Caballero *et al*.^28^ demonstrated by fractionation of the colonic microbiota of ampicillin-treated mice that colonisation resistance against vancomycin-resistant *Enterococcus faecium* requires bacterial cooperation of four commensal species: two Bacteroidales ampicillin resistant (*Bacteroides sartorii* and *Parabacteroides distasonis*) that allow engraftment of two ampicillin sensitive Firmicutes (*Clostridium bolteae* and *Blautia producta*). *B*. *producta* was shown to directly inhibit VRE growth and to cooperate with *B*. *sartorii*, *P*. *distasonis* and *C*. *bolteae* for intestinal colonisation to mediate VRE clearance. Similarly, cooperative interaction between a β-lactamase producing strain of *B*. *thetaiotaomicron* and the microbiota prevented overgrowth of VRE^54^. On top of that, correlation of *Bacteroides* spp. preservation during Fidaxomicin treatment and reduced risk of acquisition and overgrowth of VRE further support *Bacteroides* as contributors of anti-VRE effect^55,56^. Thus, it is tempting to hypothesise a cooperative mechanism involving the *L*. *paracasei* CNCM I-3689 strain and members of the Bacteroidales order. In the case of mice, the transfer of a particular bacterium due to coprophagy may promote Bacteroidetes recovery. A reductionist approach in simplified ecosystems in gnotobiotic mice should help to identify the keystone species of anti-VRE effect. A limitation of the current study is that mice were co-housed in a single cage for all experiments in a ratio of 4–5 mice per cage. Although cages were changed every three days, this set up may raise the question of a cage effect and of the impact of coprophagy on our findings. One outlier mouse in a cage could influence the microbiota recovery (or the VRE levels as discussed previously). However, regarding the effect on the resilience at phyla level, previous work supports that the cage effect and coprophagy has more effect on lower taxonomic levels^57^. To overcome this confounding factor daily cage change and less mice housed per cage will be used for future experiments.

Mechanisms controlling intestinal expansion of VRE may involve microbiota-mediated inhibition through the production of inhibitory metabolites or nutrient competition, and/or host immune functions modulated by the microbiota^22,58^. Colonisation resistance against various pathogens associates with restoration of bacterial metabolites^48,59,60^. Short chain fatty acids are microbial metabolites that have been shown to exert various anti-pathogenic effects, such as direct growth inhibition or reduction of oxygen availability through the host response^22,61^. Secondary bile acids synthesised by the gut microbiota have a strong antibacterial activity^62^ and contribute to inhibit *C*. *difficile* intestinal colonisation^60^. The observed trend of higher level of lithocholate and possibly propionate in some trials upon *L*. *paracasei* CNCM I-3689 supplementation may contribute to anti-VRE effect and derive from improved recovery of the microbiota, including Bacteroidetes known to be propionate producers^63^. We could speculate a direct or indirect role of Bacteroidetes in the recovery of propionate and/or secondary bile salt metabolism by the microbiota. Host response analysis provides us clues on the late mechanism against VRE. In accordance with a previous study, we found that *L*. *paracasei* CNCM I-3689 induces colonic epithelial cell proliferation^64^. The rate of intestinal renewal has been shown to provide an important intrinsic defense system as it probably participates to a better reduction of pathogens^65^. Notably, the supplementation of *L*. *paracasei* CNCM I-3689 is associated with an increase of Camp (LL-37 in human), and a decrease of Il12. Cathelicidin LL-37 has potent antimicrobial effect against *E*. *faecalis*^66^ indicating a potential role for an antimicrobial activity against *E*. *faecalis* in the gut. No effect of *L*. *paracasei* CNCM I-3689 on *camp* nor *il12a* expression was observed in a previous study performed on a gnotobiotic model^31^, arguing for an indirect effect of *L*. *paracasei* CNCM I-3689 on the ileal expression of *camp* or *il12a* genes. It is tempting to postulate that *L*. *paracasei* CNCM I-3689 may have an indirect effect on intestinal host response through improved recovery of Bacteroidetes by potentially creating a more favourable niche. Interestingly, Koh and collaborators demonstrated that Bacteroidetes namely *Bacteroides thetaiotaomicron* stimulates LL-37/CRAMP intestinal expression that can promote colonisation resistance against *Candida albicans*, another opportunistic pathogen in immunocompromised patients^67^. Also, intestinal expansion of VRE is controlled by RegIIIg, which ileal expression is induced by gram-negative commensal bacteria outnumbered by Bacteroidetes^68,69^. Our study highlights that a better understanding of the role of the microbiota on the production of ileal antimicrobial peptides and bacterial metabolites is needed to better control intestinal expansion of VRE. Despite the complexity of the pathobiome interplay between the microbiota, the host and the pathobiont, this preclinical study reveals the strain *L*. *paracasei* CNCM I-3689 improves VRE-reduction and resilience of some members of the gut microbiota (mainly Bacteroidetes) in an antibiotic-induced dysbiosis. Preclinical and clinical studies are required to corroborate and elucidate the underlying anti-VRE mechanisms of *L*. *paracasei* CNCM I-3689 and to assess its efficacy in human.

## Material and Methods

### Bacterial strains and growth

*E*. *faecalis* V583 strain^70^ was grown in M17 supplemented with 0.5% glucose (M17G) at 37 °C under static conditions. Strains *L*. *paracasei* CNCM I-3689 and *L*. *rhamnosus* CNCM I-3690 were grown in MRS at 37 °C in static liquid medium or in anaerobic jars on plates. Bacterial inocula were prepared using bacteria collected by centrifugation 1 h after reaching stationary phase. Bacteria were washed twice with 0.9% saline solution and stored as dry frozen pellets at −80 °C^35^. Before inoculation, a frozen bacterial pellet was suspended in a saline solution and serial diluted before plating on M17G or MRS agar to determine the bacterial count and adjust concentration at 10^9^ CFU of *Lactobacillus* strains in 0.1 ml for administration to mice.

### *In vivo* experimental design

All animals were handled in strict accordance with good animal practice as defined by the local animal welfare bodies (Unité IERP, INRA Jouy-en-Josas, France). Animal work was carried out under the authority of license issued by the national Direction des Service Vétérinaires (accreditation number A78–187 to LR-G), and approved by COMETHEA, the appropriate local ethic committee (authorisation number 12/081). CF-1 mice originally purchased from Envigo (Indianapolis, USA) were raised under specific pathogen-free conditions at the CDTA-CNRS (Orléans, France). Mouse experiments were performed using male CF-1 mice aged 6–8-weeks and 4 to 8 mice per group. A maximum of 5 mice were housed in each cage and were fed with autoclaved food and water *ad libitum*. Mice received a daily dose of 10^9^ CFU of *L*. *paracasei* CNCM I-3689 or *L*. *rhamnosus* CNCM I-3690 strain in 0.1 ml of 0.9% NaCl (saline solution) by orogastric inoculation using a feeding tube (Ecimed). Animals from the control group received 0.1 ml of saline solution. After one week of supplementation, a dose of 1.4 mg/day of clindamycin was administered subcutaneously daily for three days. In experiments referred as trials 1, 2 and 3 mice received 10^10^ colony-forming units (CFU) of *E*. *faecalis* VRE strain V583 by orogastric inoculation one day after stopping the clindamycin treatment (D10).

Stool samples were collected at D0, D7, D10 or D11, D14, D18 and D21. Fecal samples collected for 16S rRNA gene survey analysis of the whole microbiota were stored at −80 °C. Feces (from 50 to 100 mg/mice) kept at 4 °C were treated within 3 hours after sampling and processed at room temperature. From this stage, all steps were performed in sterile conditions. Samples were weighted and suspended at a dilution of 10^−1^. An adjusted volume of peptone water was added according to the weight (eg. 900 µl for 100 mg, 450 µl for 50 mg). A volume of 100 µl of the suspension (dilution -1) was used to perform decimal dilutions in peptone water until 10^−8^. Total enterococci count was monitored by plating onto BEA, and total lactobacilli on MRS medium at 37 °C under anaerobic condition (Gas pack) for 48 h. The number of *E*. *faecalis* V583 was followed by plating onto BEA supplemented with vancomycin at 6 µg/ ml. All mice were euthanized at the end of the experiment. Small intestine and colon tissues were recovered and immediately stored in liquid nitrogen or placed into paraformaldehyde solution 4% for further RNA extraction or histological and immunochemistry analysis, respectively.

### Analysis of fecal microbiota by 454 pyrosequencing

DNA of fecal samples was extracted following Godon *et al*. protocol^71^. Control quality of DNA samples was assessed according to Life Sequencing instructions (Lifesequencing S.L., Valencia, Spain). The 16S rRNA genes were sequenced by Life Sequencing based on the analysis of the V3-V5 region^72^ using a 454 Life Sciences GS FLX + instrument (Roche).

Bioinformatic analyses were performed using QIIME v.1.9^73^. Data from Life sequencing were assigned to the samples after filtering according to the following criteria: size between 200 and 1000 nt, quality above 25 over a 50 base pairs window, no mismatch authorised in primers and barcode sequences, and absence of polymers larger than 6 nt. Remaining reads were clustered into Operational Taxonomic Units (OTUs) defined at 97% identity using Usearch and representative sequences for each OTU were aligned and taxonomically assigned using Silva database (version 111). For alpha and beta diversity, samples were rarefied to 1500 sequences per sample. Alpha-diversity (that measures diversity within samples) was assessed using rarefaction curves for Shannon index, and numbers of observed OTUs. Beta diversity between samples was performed on weighted and unweighted Unifrac and Bray-Curtis distances using 1500 reads.

### *In vitro* interaction in liquid culture

To assess possible effect of *L*. *paracasei* CNCM I-3689 strain on *E*. *faecalis* growth *in vitro*, the supernatant of an overnight culture of *L*. *paracasei* CNCM I-3689 was recovered by centrifugation at 15,000 g for 15 min. The pH of the supernatant was adjusted to reach approximately 7 (6.8–7.2) with NaOH. Then, the supernatant was filtrated on 0.2 µm. Five ml of filtrated supernatant were added to 5 ml of M17G. The resulting conditioned medium was inoculated with 100 µL of an overnight culture of *E*. *faecalis* V583. *E*. *faecalis* growth was compared to growth in a control tube of equal volumes of MRS and M17G. *E*. *faecalis* V583 growth was monitored by measuring OD at 600 nm and plating.

### Quantification of expression of selected mRNAs in the small intestine

A custom-designed TaqMan Low Density Array (TLDA) card was configured into 8 identical 48-gene sets. The list of genes analysed is given in Supplementary Table S3.

Total RNAs were extracted from small intestine fragments using the mirVana miRNA isolation Kit (Life Technologies) according to the recommendations of the manufacturer. RNA quality was assessed with the Agilent 2100 Bioanalyzer (Agilent Technologies). RNA integrity number (RIN) was equivalent among groups and was of 8.1 ± 0.3 (n = 16). Complementary DNA (cDNA) was generated by reverse transcription (RT) with random hexamers and the High-Capacity cDNA Reverse Transcription d’Applied. cDNA, corresponding to 50 ng of starting RNA, was mixed with TaqMan Universal PCR Master Mix (Applied Biosystems, Inc.) and loaded into one of the eight fill ports on the TLDA microfluidic card. The cards were briefly centrifuged for 1 min at 1300 × g to distribute the reaction mix to each of the reaction wells and were then sealed to prevent well-to-well contamination. PCR amplifications were performed on a QuantStudio Real Time PCR Detection System (Applied Biosystems) under the following thermal-cycling conditions: 95 °C for 10 minutes followed by 40 cycles of 95 °C for 15 seconds and 60 °C for 1 minute. Cycle threshold (Cq) values were extracted using Real-Time qPCR Analysis software (Applied Biosystems) [using Symphony™ Suite analysis software (www.lifetechnologies.com)]. The fold-change (Rq or relative quantification) in the expression of target genes between Lactobacilli-supplemented and control mice were calculated using the comparative 2−ΔΔCq method^74,75^. Results obtained were normalised to those for the Ubiquitin gene (Mm01201237_m1) as internal control and compared with the mean target gene expression in NaCl treated mice. Further validation of data obtained from TLDA was carried out using single Taqman assays. *camp* and *Il-12a* mRNA were analysed using Applied Biosytems designed Taqman assays Mm00438285_m1 and Mm00434165_m1 respectively. The assay Mm99999915_g1 corresponding to *gapdh* (glyceraldehyde-3-phosphate dehydrogenase) was used as reference gene in single assay measurement.

### Histology and immunochemistry

Samples were fixed in 4% paraformaldehyde (24 h, 4 °C), dehydrated, and embedded in paraffin, according to standard histological protocols. Sections (5 μm) of tissues were mounted on SuperFrost Plus slides (Thermo Fisher, Waltham, MA, USA). After dewaxing and rehydratation, sections were heated at 97 °C in 10 mM citrate buffer (pH 6.0) for 40 min. Nonspecific binding was blocked using protein block serum-free (X0909; DakoCytomation) for 1 h at room temperature. Sections were incubated with primary antibodies diluted in antibody diluent (S3022; DakoCytomation) overnight at 4 °C. The primary antibodies used were anti-Z0–1 (1:500; LifeTech), anti-claudin-1 (1:500; InVitrogen), anti-claudin-2 (1:500; InVitrogen), anti-PCNA (1:1000; GeneTex) and anti-Ki67 (1:50, DakoCytomation). Nuclei were stained with Hoechst before mounting the slides using Fluorescent Mounting Media (Dako). Sections were scanned using a Pannoramic Scan digital slide scanner (3DHistech) and analysed using digital slide scanner Pannoramic scan (3Dhistech). For Ki67 and PCNA 10 crypts were measured per mice and per cut.

### Short Chain Fatty Acids (SCFA) analysis

SCFA (acetic, propionic and butyric acid) were analysed and concentrations were determined with gas chromatography (Nelson 1020, Perkin‐Elmer, St Quentin en Yvelines, France) as described previously^76^. Results are expressed in relative percentage of each SCFA.

### Quantification of cecal bile acids

Cecal contents (5 controls and 8 from *L*. *paracasei* treated mice) from trial 3 were used to quantify primary and secondary bile acids. Bile acids analysis was performed at Bioaster (Lyon, France). Samples were prepared from 50 to 60 mg of −80 °C frozen cecal content using aqueous methanol extraction process. Bile acids extracts were analysed by LC-MS/MS on a triple quadrupole Thermo Quantum Ultra (SN: TQU00665) combined to a Dionex Ultimate 3000 HPLC system (SN: 8074045 & 8087183). Samples were separated on a C18 column (2,7 μM, 150 × 2,1 mm) from Ascentis Express using a methanol/water gradient containing 5 mM ammonium acetate and 0,012% formic acid. All bile acid standards: Lithocholic acid, Chenodeoxycholic acid, Deoxycholic acid, Ursodeoxycholic acid, Cholic acid, Glycodeoxycholic acid, Glycoursodeoxycholic acid, Glychenodeoxycholic acid, Glychocolic acid, Taurolithocholic acid, Taurodeoxycholic acid, Taurocholic acid, were bought from Sigma Aldrich. Results of bile salt quantification were expressed in peak area, corrected for sample weight.

### Statistical analysis

Comparison of kinetics of VRE in control and *L*. *paracasei* groups was performed separately on each trial. The difference at each time point was tested by Mann-Whitney test. The P-values p_11_, p_14_, p_16_, p_18_, p_21_ corresponding to days 11, 14, 16, 18, 21 were gathered using Fisher’s combined probability method: the statistic S = −Σ log(p_t_) equal to the sum of the inverse of the log-transformed gets larger as soon as a significant difference between groups is observed at one time point. A permutation p-value was then computed by random reallocation of group labels (control and *L*. *paracasei*).

The PERMANOVA (Permutational multivariate analysis of variance) test implemented in the function adonis2 of the R-package vegan was used to test global differences in microbiota composition (a) between D0 and D7 separately on control and *L*. *paracasei* groups grouping mice from the five trials, (b) between D0 and D21 separately on each trial, (c) between D0 and D21 separately for control and *L*. *paracasei* groups and for trials with and without VRE inoculation (trials 1 and 3 and trials 4 and 5, respectively). In order to erase individuals effect, in each comparison between two groups, only mice with measurements in both groups were considered. PERMANOVA test is based on prior calculation of a matrix of two-by-two distance between all pairs of samples. In our analysis, we considered the following distances: weighted- and unweighted-Unifrac distance, and Bray-Curtis distance based on OTU. These three distances led to similar conclusions.

The difference of abundances between control and *L*. *paracasei* groups for each phylum was assessed using two distinct statistical tests: Mann-Whitney test for phyla with less than 10% of zeros and a Fisher presence/absence test for phyla with more than 50% of zeros. For phyla, which proportion of zeros ranged between 10% and 50%, the minimum of the two p-values was considered.

Short chain fatty acid, bile salts and host response data were analysed by the Mann-Whitney test (GraphPad 4.03).

## Electronic supplementary material

Supplementary information
