# Supplementary material for: Lactobacillus paracasei CNCM I-3689 reduces vancomycin-resistant Enterococcus persistence and promotes Bacteroidetes resilience in the gut following antibiotic challenge
Source: Sci Rep. 2018 Mar 23;8:5098. doi: 10.1038/s41598-018-23437-9 (PMC5865147; doi:10.1038/s41598-018-23437-9)
Supplement: Supplementary file 1 — Supplementary information [file 41598_2018_23437_MOESM1_ESM.pdf]

Lactobacillus paracasei CNCM I-3689 reduces vancomycin-resistant _Enterococcus_ persistence and promotes Bacteroidetes resilience in the gut following antibiotic challenge

Laureen Crouzet[1#°], Muriel Derrien[2#], Claire Cherbuy[1], Sandra Plancade[3], Mélanie Foulon[1], Benjamin Chalin[1], Johan E.T. van Hylckama Vlieg[2§], Gianfranco Grompone[2¤], Lionel Rigottier-Gois[1], Pascale Serror[1*]

[1] Micalis, INRA, AgroParisTech, Université Paris-Saclay, 78350 Jouy-en-Josas, France
[2] Danone Nutricia Research, F-91120 Palaiseau, France
[3] Maiage, INRA, Université Paris-Saclay, 78350 Jouy-en-Josas, France

# Laureen Crouzet and Muriel Derrien contributed equally to this work.

Current address:
° Medis, INRA Clermont-Ferrand-Theix, 63122 Saint-Genès-Champanelle, France
¤ Instituto Nacional de Investigación Agropecuaria, Montevideo, Urugay
§ Chr. Hansen AS, Hoersholm, Denmark

* For correspondence. E-mail: pascale.serror@inra.fr

Supplementary Table S1. Fecal DNA samples used for 16S sequencing.

**Trial**	**Inoculation of *E. faecalis* V583**	**Groups and time points**	
**1**	Yes	*L. paracasei* (n=21)<br>D0 (n=5)<br>D7 (n=4)<br>D10 (n=3)<br>D11 (n=4)<br>D21 (n=5)	NaCl 0.9% (n=19)<br>D0 (n=5)<br>D7 (n=3)<br>D10 (n=3)<br>D11 (n=5)<br>D21 (n=3)
**2**	Yes	*L. paracasei* (n=21)<br>D0 (n=5)<br>D7 (n=3)<br>D10 (n=3)<br>D11 (n=5)<br>D21 (n=5)	NaCl 0.9% (n=18)<br>D0 (n=4)<br>D7 (n=3)<br>D10 (n=3)<br>D11 (n=4)<br>D21 (n=4)
**3**	Yes	*L. paracasei* (n=15)<br>D0 (n=7)<br>D21 (n=8)	NaCl 0.9% (n=10)<br>D0 (n=5)<br>D21 (n=5)
**4**	No	*L. paracasei* (n=21)<br>D0 (n=5)<br>D7 (n=3)<br>D11 (n=5)<br>D14 (n=3)<br>D21 (n=5)	NaCl 0.9% (n=16)<br><br>D7 (n=3)<br>D11 (n=5)<br>D14 (n=3)<br>D21 (n=5)
**5**	No	*L. paracasei* (n=21)<br>D0 (n=5)<br>D7 (n=3)<br>D11 (n=5)<br>D14 (n=3)<br>D21 (n=5)	NaCl 0.9% (n=21)<br>D0 (n=5)<br>D7 (n=3)<br>D11 (n=5)<br>D14 (n=3)<br>D21 (n=5)

Supplementary Table S2. Phylum relative abundance (Values are mean percentage read number±SEM) in the gut microbiota in control and *L. paracasei* CNCM I-3689-supplemented mice over time in the presence of strain *E. faecalis* V583 inoculated at D11.

	Control					*L. paracasei* CNCM I-3689				
Day	0	7	10	11	21	0	7	10	11	21
**Trial 1** (n=3 to 5)										
Actinobacteria	0.7±0.2	1.1±0.2	0±0	0±0	0±0	0.8±0.2	0.5±0.1	0±0	0±0	0±0
Bacteroidetes	38±4.7	22.5±3.2	0±0	0.1±0	0.6±0	28±6.1	47.7±5.2	0±0	3.2±3.2*	5.6±1.2
Deferribacteria	0.2±0.1	1.1±0.5	0±0	0±0	0±0	1.3±0.4	0.5±0.2	0±0	0±0	0±0
Firmicutes	60±4.8	73.7±2.8	2.4±0.9	41.2±7.7	98.2±0.3	68.3±5.8	50±4.9	0.8±0.2	31.3±7.3	91.6±1.1
Other	0.0±0.0	0±0	0±0	0±0	0±0	0.1±0.1	0.3±0.1	0±0	0±0	0.4±0.4
Proteobacteria	0.5±0.2	1.1±0.4	97.6±0.9	58.7±7.8	1.2±0.3	1.1±0.5	0.5±0.2	99.1±0.2	65.4±7.2	2.3±0.6
**Trial 2** (n=3 to 5)										
Actinobacteria	0.8±0.1	1.1±0.2	0±0	0±0	0.1±0.1	1.2±0.2	0.6±0	0±0	0±0	0.3±0.2
Bacteroidetes	49.5±4.5	53.5±0.5	0±0	0±0	1±0.7	48.8±6.3	22.7±2.4	0±0	0.1±0.1	0.2±0.1
Deferribacteria	0.4±0.1	0.1±0.1	0±0	0±0	0±0	0.1±0	1.5±0.5	0±0	0±0	0±0
Firmicutes	47.4±4.7	42.5±0.9	22.2±4.1	39.1±10.5	97.3±1	47.2±6.8	73.8±2.5	28.1±7.6	78.6±2.8	98.4±0.3
Other	0.3±0.1	0.6±0.1	0±0	0±0	0.1±0.1	0.5±0.2	0.3±0.2	0±0	0±0	0.2±0.1
Proteobacteria	1.3±0.4	1.7±0.7	77.7±4	60.8±10.5	1.1±0.7	1.8±0.5	0.8±0.2	71.4±7.7	21±2.9	0.7±0.2
**Trial 3** (n=4 to 8)										
Actinobacteria	1.2±0.4	nd¤	nd	nd	0.3±0.1	0.2±0.1	nd	nd	nd	0.2±0.1
Bacteroidetes	41.3±6	nd	nd	nd	19.8±15.5*	27.9±3.3	nd	nd	nd	27±5.6
Deferribacteria	0.3±0.1	nd	nd	nd	0±0	0.4±0.1	nd	nd	nd	0±0
Firmicutes	55.6±5.7	nd	nd	nd	78.4±15.7*	70.1±3.2	nd	nd	nd	71.6±5.8
Other	0.2±0.1	nd	nd	nd	0.3±0.1	0.2±0.1	nd	nd	nd	0±0
Proteobacteria	0.6±0.1	nd	nd	nd	0.4±0.2	0.4±0.1	nd	nd	nd	0.2±0
**Trial 4** (n=3 to 5)										
Actinobacteria	nd	0.3±0.2	0±0	0±0	0.3±0.2	0.4±0.2	0.3±0	0±0	0±0	0.1±0
Bacteroidetes	nd	32±12.2	0.1±0.1	0±0	0±0	33.4±8.6	20.9±8.8	0±0	0±0	20.9±9.2*
Deferribacteria	nd	0.3±0.2	0±0	0±0	0±0	1±0.6	1.2±1.1	0±0	0±0	0±0
Firmicutes	nd	66.3±12.4	13.8±7.8	45.6±12.7	98.8±0.5	63.7±8.2	76.2±7.7	18.2±8.2	62.5±13	78.6±9.4
Other	nd	0±0	0±0	0±0	0±0	0.1±0.1	0.2±0.1	0±0	0±0	0±0
Proteobacteria	nd	0.4±0.1	86±7.7	54.2±12.8	0.8±0.3	0.7±0.2	0.5±0.3	81.7±8.2	37.1±12.8	0.2±0.1
**Trial 5** (n=3 to 5)										
Actinobacteria	0.2±0.1	0.2±0	0±0	0±0	0±0	0.1±0	0.2±0.1	0±0	0.2±0.2	0±0
Bacteroidetes	28.2±10.4	27±2.7	0±0	0±0	0±0	71.5±7.6	61.6±2.5	0±0	0.3±0.1	8.7±3.1*
Deferribacteria	1±0.6	0.4±0.1	0±0	0±0	0±0	0.1±0	0.2±0	0±0	0±0	0±0
Firmicutes	69.4±10	70.9±2.6	20.1±4.3	81.9±8.7	95±1.5	25.7±6.6	36.7±2	21.1±3.5	80.3±10.6	88.5±4
Other	0±0	0±0	0±0	0±0	0.1±0.1	1.6±1.2	0.2±0.2	0±0	0.3±0.3	0.1±0.1
Proteobacteria	0.4±0.2	0.7±0.4	79.8±4.3	18.1±8.7	4.9±1.6	0.4±0.1	0.6±0.2	78.6±3.7	18.8±11.1	2.6±1

* One outlier value out.

¤ Not determined.

Supplementary Table S3. PERMANOVA p-values for the difference of microbiota composition between D0 and D7 separately for control and *L. paracasei* mice from trials 1 to 5. Three distances were used for PERMANOVA test: unweighted and weighted Unifrac, and Bray-Curtis distance calculated from OTU abundances.

Group	Uw-Unifrac*	W-Unifrac*	BC OTU°
Control	0.46	0.61	0.74
L. paracasei	0.77	0.89	0.66

* Uw- and W-Unifrac for unweighted and weighted Unifrac, respectively.

° BC for Bray-Curtis distance.

Supplementary Table S4. PERMANOVA p-values for the difference of microbiota composition between D0 and D21, performed separately on mice inoculated (trials 4 and 5) or not (trials 1and 3) with *E. faecalis* V583 and supplemented with *L. paracasei* (*L. paracasei* group) or without (control group). Three distances were used for PERMANOVA test: unweighted and weighted Unifrac, and Bray-Curtis distance calculated from OTU abundance. Trial 2 was not included because no anti-VRE effect was observed in this trial.

Trials - group	uw-Unifrac*	w-Unifrac*	BC OTU°
1 and 3 - control	0.001	0.002	0.001
1 and 3 - *L. paracasei*	0.001	0.009	0.001
4 and 5 - control	0.008	0.009	0.006
4 and 5 - *L. paracasei*	0.001	0.001	0.001

* Uw- and W-Unifrac for unweighted and weighted Unifrac, respectively.

° BC for Bray-Curtis distance.

Supplementary Table S5. Fisher and Mann-Whitney p-values for the difference of microbiota composition at the phylum level from mice supplemented with *L. rhamnosus* (n=8) or with NaCl 0.09% (n=5) at D21.

	Actinobacteria	Bacteroidetes	Cyanobacteria	Firmicutes	Proteobacteria	Tenericutes
Proportion of zero	0.46	0	0.69	0	0.38	1
Mann-Whitney*	0.76	0.62	0.53	0.52	0.40	NA
Fisher*	0.59		1		0.56	NA

* p-values were adjusted using the Benjamini–Hochberg correction.

Supplementary Table S6. Transcriptomic analysis of a selection of 42 host genes upon *L. paracasei* CNCM I-3689-supplementation.

Group	Gene	Fold change expression ± SEM in *L. paracasei* compared to control mice	p-value
Control	Ang4-Mm03647554_g1	1.22 ± 0.66	0.83
L. paracasei	Ang4-Mm03647554_g1	1.27 ± 0.85	
Control	Camp-Mm00438285_m1	0.92 ± 0.14	0.05
L. paracasei	Camp-Mm00438285_m1	1.51 ± 0.29	
Control	Ccl2-Mm00441242_m1	0.58 ± 0.28	0.15
L. paracasei	Ccl2-Mm00441242_m1	1.06 ± 0.59	
Control	Ccl9-Mm00441260_m1	1.38 ± 0.43	0.32
L. paracasei	Ccl9-Mm00441260_m1	1.31 ± 0.69	
Control	Ccnd1-Mm00432359_m1	1.27 ± 0.29	0.43
L. paracasei	Ccnd1-Mm00432359_m1	1.04 ± 0.33	
Control	Ccne1-Mm00432367_m1	1.00 ± 0.11	0.26
L. paracasei	Ccne1-Mm00432367_m1	0.97 ± 0.39	
Control	Cx3cr1-Mm00438354_m1	0.80 ± 0.17	0.19
L. paracasei	Cx3cr1-Mm00438354_m1	1.24 ± 0.59	
Control	Defa-rs1-Mm00655850_m1	1.96 ± 1.02	0.95
L. paracasei	Defa-rs1-Mm00655850_m1	1.91 ± 1.10	
Control	Duox1-Mm01328685_m1	1.03 ± 0.33	0.12
L. paracasei	Duox1-Mm01328685_m1	1.46 ± 0.59	
Control	Foxo1-Mm00490672_m1	0.91 ± 0.06	0.79
L. paracasei	Foxo1-Mm00490672_m1	0.88 ± 0.17	
Control	Foxp3-Mm00475162_m1	0.77 ± 0.29	0.23
L. paracasei	Foxp3-Mm00475162_m1	0.53 ± 0.38	
Control	Fut2-Mm00490152_s1	1.19 ± 0.66	0.95
L. paracasei	Fut2-Mm00490152_s1	1.02 ± 1.13	
Control	Gata3-Mm00484683_m1	1.12 ± 0.41	0.56
L. paracasei	Gata3-Mm00484683_m1	0.93 ± 0.29	
Control	Gzmc-Mm01313651_m1	1.21 ± 0.48	0.68
L. paracasei	Gzmc-Mm01313651_m1	2.05 ± 2.03	
Control	Ifng-Mm00801778_m1	2.69 ± 1.24	0.22
L. paracasei	Ifng-Mm00801778_m1	1.19 ± 0.74	
Control	Il10-Mm00439614_m1	0.80 ± 0.31	0.49
L. paracasei	Il10-Mm00439614_m1	0.61 ± 0.26	
Control	Il12a-Mm00434165_m1	0.99 ± 0.30	0.07
L. paracasei	Il12a-Mm00434165_m1	0.23 ± 0.09	
Control	Il13-Mm00434204_m1	0.99 ± 0.35	0.74
L. paracasei	Il13-Mm00434204_m1	1.06 ± 0.21	
Control	Il18-Mm00434225_m1	1.08 ± 0.48	0.13
L. paracasei	Il18-Mm00434225_m1	1.88 ± 0.97	
Control	Il1b-Mm00434228_m1	0.78 ± 0.19	0.63
L. paracasei	Il1b-Mm00434228_m1	0.71 ± 0.28	

Continued on following page

Supplementary Table S6 - *Continued*

Group	Gene	Fold change expression ± SEM in *L. paracasei* compared to control mice	p-value
Control	Il23a-Mm01160011_g1	0.85 ± 0.32	0.14
L. paracasei	Il23a-Mm01160011_g1	1.44 ± 0.59	
Control	Il6-Mm00446190_m1	0.46 ± 0.30	0.77
L. paracasei	Il6-Mm00446190_m1	0.50 ± 0.27	
Control	Mki67-Mm01278617_m1	1.16 ± 0.31	0.71
L. paracasei	Mki67-Mm01278617_m1	1.22 ± 0.66	
Control	Myd88-Mm00440338_m1	1.08 ± 0.13	0.37
L. paracasei	Myd88-Mm00440338_m1	1.17 ± 0.18	
Control	Nod2-Mm00467543_m1	1.19 ± 0.42	0.43
L. paracasei	Nod2-Mm00467543_m1	1.77 ± 0.95	
Control	Pla2g2a-Mm00448160_m1	1.22 ±0.32	0.18
L. paracasei	Pla2g2a-Mm00448160_m1	1.63 ± 0.60	
Control	Prf1-Mm00812512_m1	1.64 ± 0.63	0.11
L. paracasei	Prf1-Mm00812512_m1	1.02 ± 0.41	
Control	Reg3b-Mm00440616_g1	1.97 ± 0.90	0.60
L. paracasei	Reg3b-Mm00440616_g1	1.83 ± 1.00	
Control	Reg3g-Mm00441127_m1	1.56 ± 0.64	0.38
L. paracasei	Reg3g-Mm00441127_m1	1.35 ± 0.91	
Control	Rorc-Mm01261022_m1	1.04 ± 0.21	0.63
L. paracasei	Rorc-Mm01261022_m1	1.16 ± 0.53	
Control	Saa3-Mm00441203_m1	0.42 ± 0.35	0.79
L. paracasei	Saa3-Mm00441203_m1	0.37 ± 0.27	
Control	Stat1-Mm00439531_m1	1.94 ± 0.86	0.87
L. paracasei	Stat1-Mm00439531_m1	2.79 ± 2.11	
Control	Stat3-Mm01219775_m1	1.2 ± 0.26	0.83
L. paracasei	Stat3-Mm01219775_m1	1.00 ± 0.29	
Control	Stat4-Mm00448890_m1	1.13 ± 0.29	0.11
L. paracasei	Stat4-Mm00448890_m1	0.69 ± 0.22	
Control	Stat6-Mm01160477_m1	1.05 ± 0.07	0.43
L. paracasei	Stat6-Mm01160477_m1	1.08 ± 0.18	
Control	Tbx21-Mm00450960_m1	0.78 ± 0.27	0.15
L. paracasei	Tbx21-Mm00450960_m1	0.49 ± 0.27	
Control	Tff3-Mm00495590_m1	1.02 ± 0.27	0.56
L. paracasei	Tff3-Mm00495590_m1	0.96 ± 0.32	
Control	Tgfb1-Mm01178820_m1	0.83 ± 0.13	0.79
L. paracasei	Tgfb1-Mm01178820_m1	0.86 ± 0.30	
Control	Tlr2-Mm00442346_m1	1.05 ± 0.32	0.87
L. paracasei	Tlr2-Mm00442346_m1	1.42 ± 1.02	
Control	Tlr4-Mm00445273_m1	1.01 ± 0.17	0.87
L. paracasei	Tlr4-Mm00445273_m1	2.05 ± 1.72	

Continued on following page

Supplementary Table S6 - *Continued*

Group	Gene	Fold change expression ± SEM in *L. paracasei* compared to control mice	p-value
Control	Tnf-Mm00443258_m1	0.68 ± 0.27	0.37
L. paracasei	Tnf-Mm00443258_m1	0.51 ± 0.13	
Control	Tslp-Mm01157588_m1	0.81 ± 0.44	0.88
L. paracasei	Tslp-Mm01157588_m1	1.01 ± 0.12	

Supplementary Table S7. Concentrations of the major SCFAs in caecal content of mice supplemented or not for 21 days with strain *L. paracasei* CNCM I-3689 and 10 days after the end of clindamycin regimen.

		**Concentrations[a] (mM ± SEM)**			
Trial[b]		Acetic acid	Propionic acid	Butyric acid	Total
1	Control	11.5 ± 2.8	0.1 ± 0.04	0.5 ± 0.1	12 ± 3.0
	L. paracasei CNCM-I 3689	12.4 ± 3.1	1.0 ± 0.3*[c]	1.0 ± 0.4	14.3 ± 3.8
4	Control	14.8 ± 2.6	1.4 ± 0.3	7.9 ± 2.6	24.1 ± 4.5
	L. paracasei CNCM-I 3689	11.4 ± 1.5	1.0 ± 0.1	5.2 ± 1.4	17.6 ± 2.8
5	Control	9.9 ± 3.0	0.3 ± 0.1	1.4 ± 0.9	11.7 ± 2.8
	L. paracasei CNCM-I 3689	9.2 ± 0.7	0.7 ± 0.1*[c]	1.3 ± 1.1	11.3 ± 1.2

[a] n=4 to 5 mice /group

[b] *E. faecalis* V583 was inoculated to mice of trial 1.

[c] Statistical analysis using Mann–Whitney test (*P value <0.05).

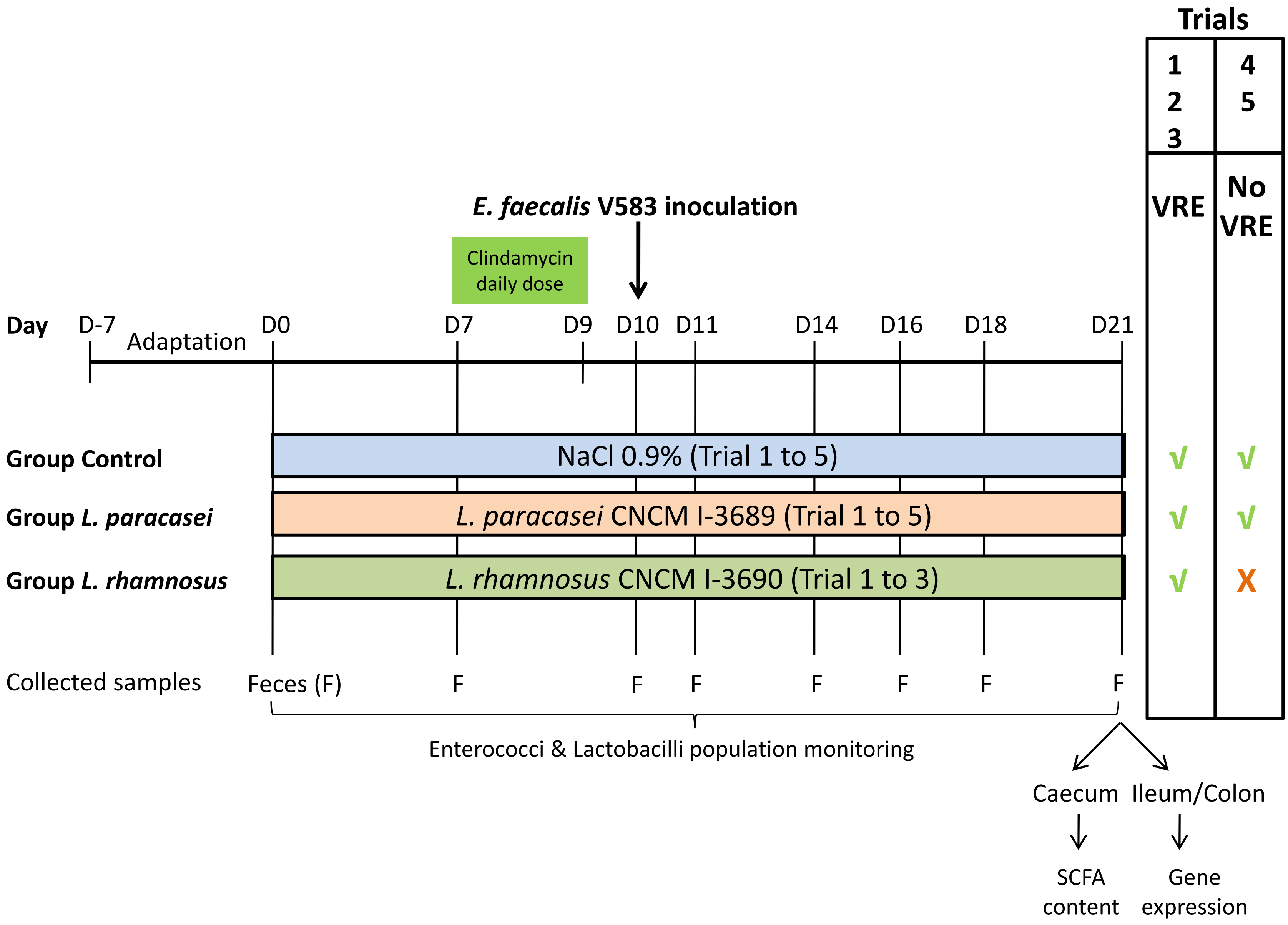

Supplementary Figure S1. Schematic representation of the experimental design of the study.

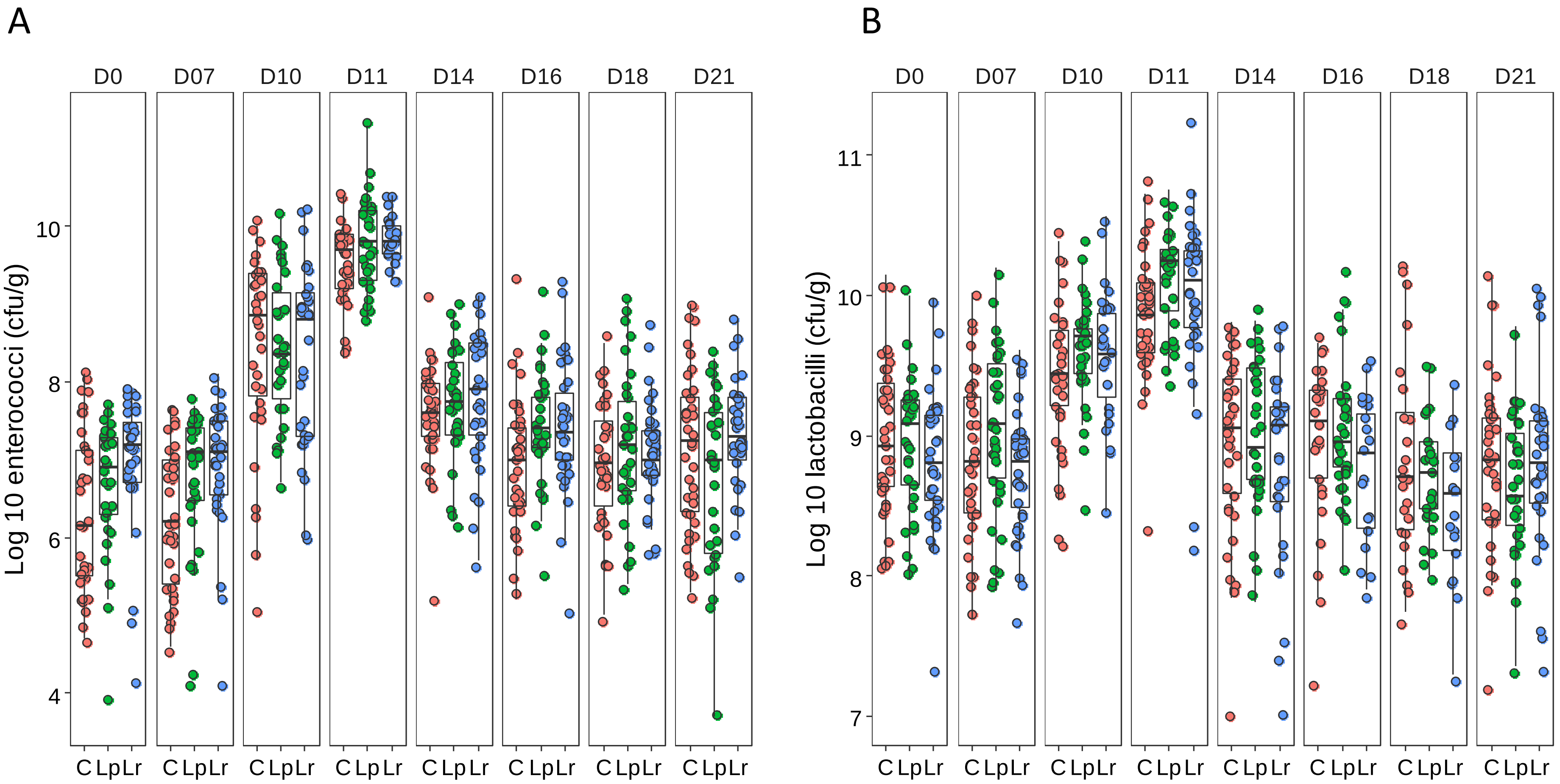

Supplementary Figure S2. Kinetics of enterococci and lactobacilli from feces in trials 1, 2 and 3. Mice supplemented with strain *L. paracasei* CNCM I-3689 (Lp, green circles) or *L. rhamnosus* CNCM I-3690 (Lr, blue circles) or not (red circles) were administered clindamycin from day 7 to day 9 and were orally inoculated with *E. faecalis* V583 at day 10. Each symbol represents the total number of colony-forming units (cfu) in feces from 15 to 20 different mice for enterococci (A) and 9 to 18 different mice for lactobacilli (B).

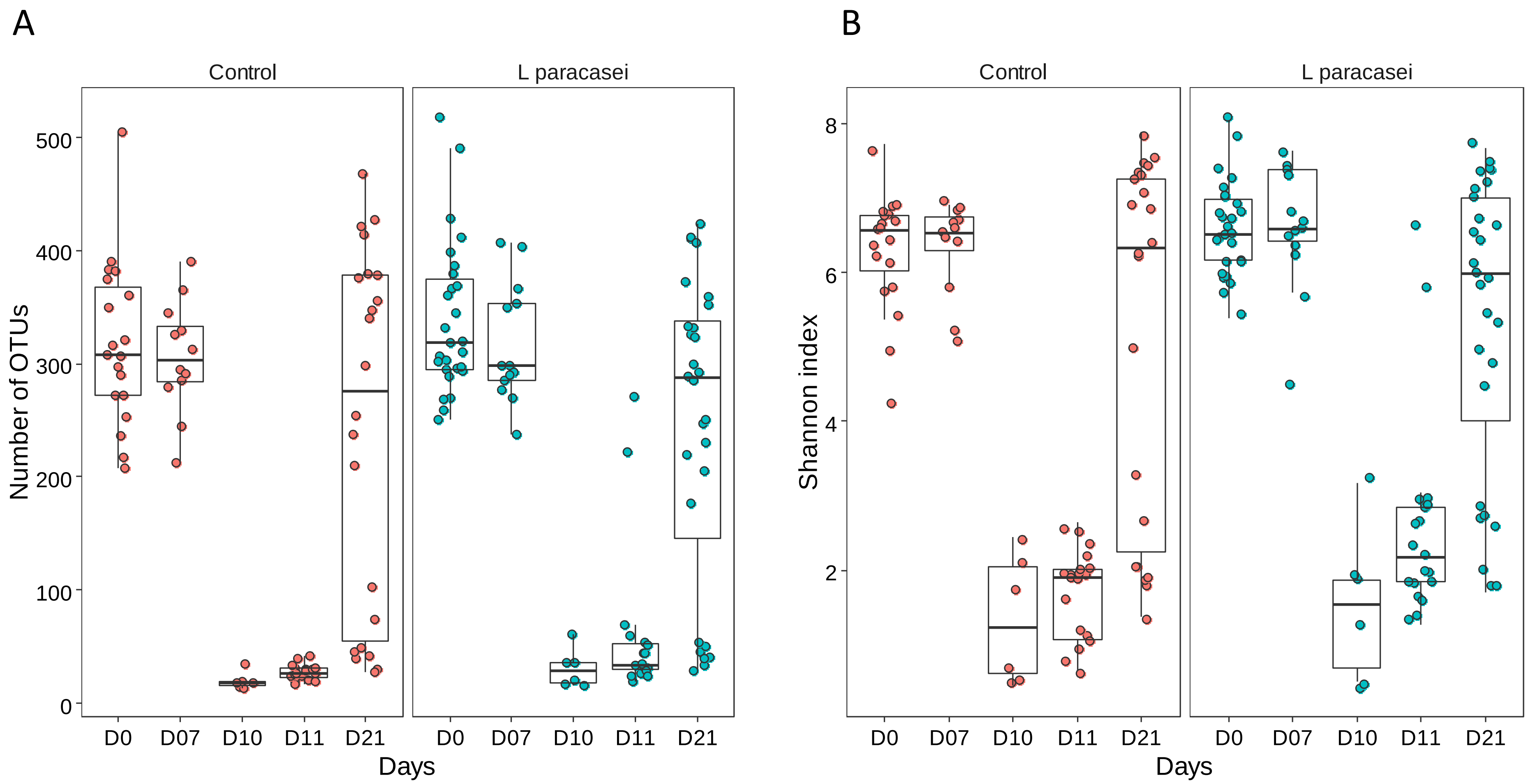

Supplementary Figure S3. **Effect of supplementation with *L. paracasei* CNCM I-3689 on the gut microbiota alpha-diversity.** Alpha-diversity was measured by number of OTUs and Shannon index in presence (Trials 1, 2 and 3) or absence (Trials 4 and 5) of *E. faecalis* V583.

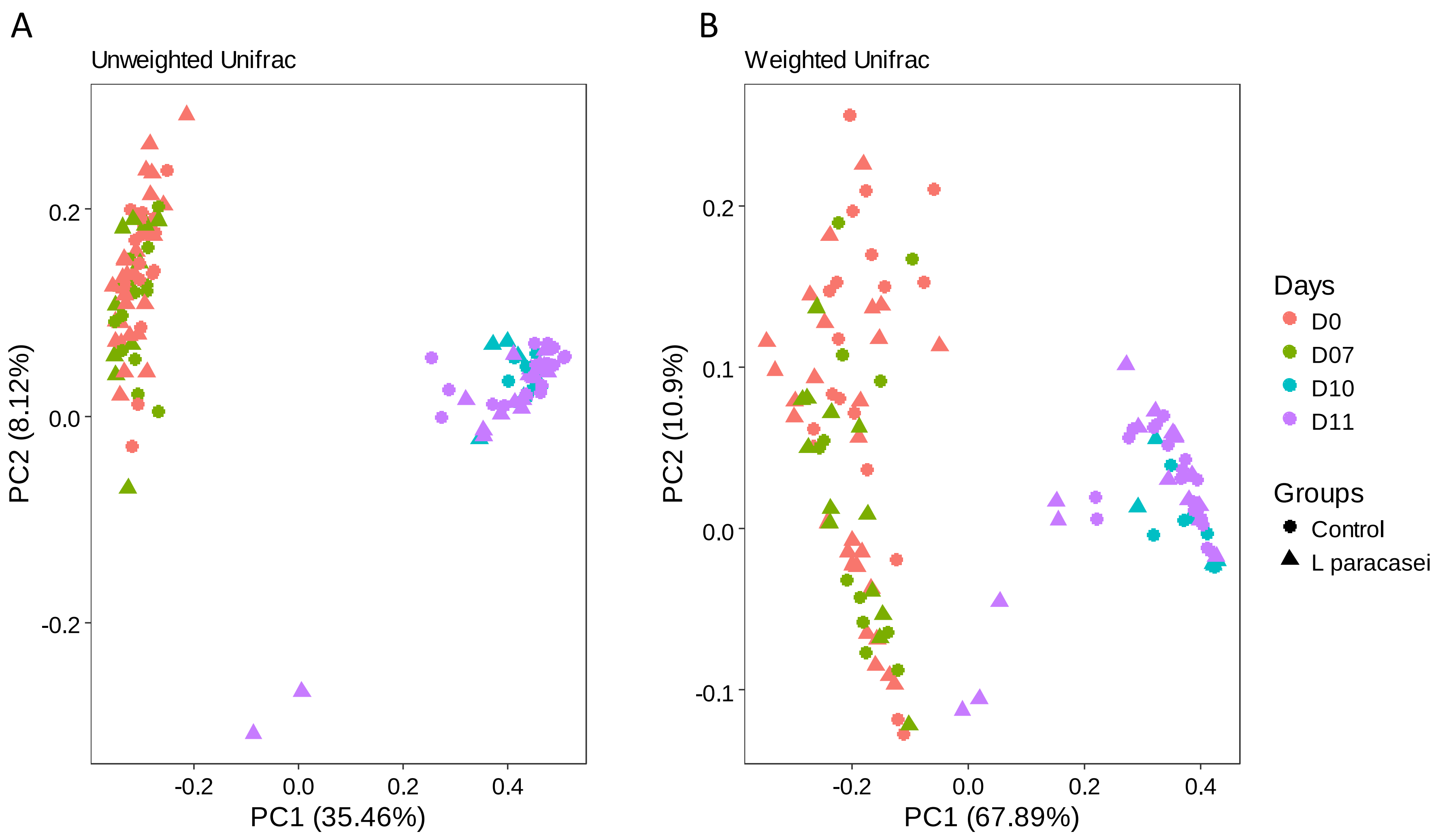

Supplementary Figure S4. ***L. paracasei* CNCM I-3689 supplementation has no major effect on eubiotic nor dysbiotic microbiota.** Principal coordinate analysis (PCoA) of gut microbiota based on unweighted (A) and weighted (B) Unifrac distances for samples from the control and *L. paracasei* CNCM I-3689 groups of the five trials at baseline (D0), 1 week after supplementation (D7), and one (D10) or two (D11) days after the cessation of the clindamycin-treatment. The score plot shows the clustering of the different samples. Samples that lie close to each other are similar with respect to the measured variables. Samples that lie diametrically opposite to each other are different from each other. The percentage of the variance explained by each axis is shown.

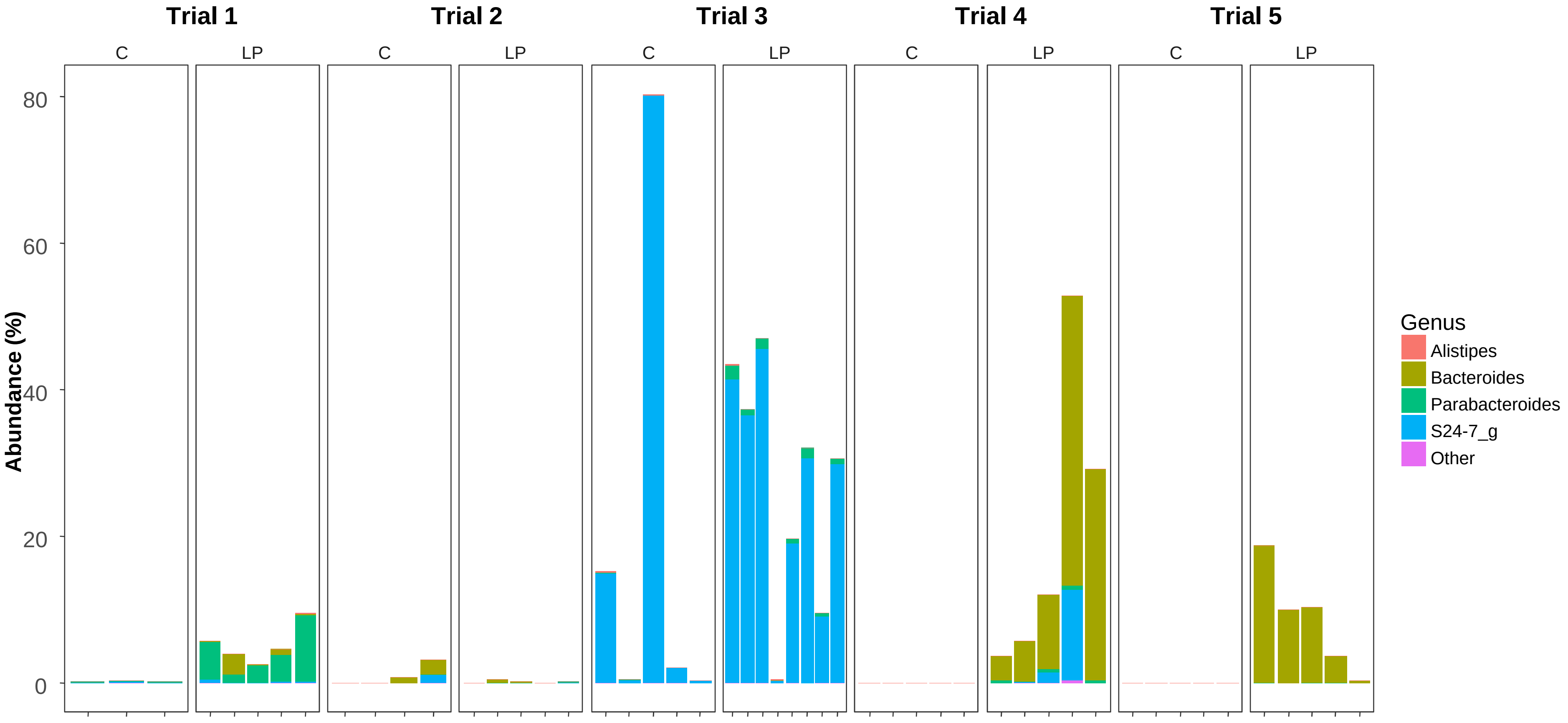

Supplementary Figure S5. Effect of supplementation with *L. paracasei* CNCM I-3689 on the recovery of the Bacteroidetes. Relative abundance of the major genera (>0.01%) of the order Bacteroidales, the only one detected from Bacteroidetes at D21 from all trials. Each bar represents the microbiota of an individual mouse.

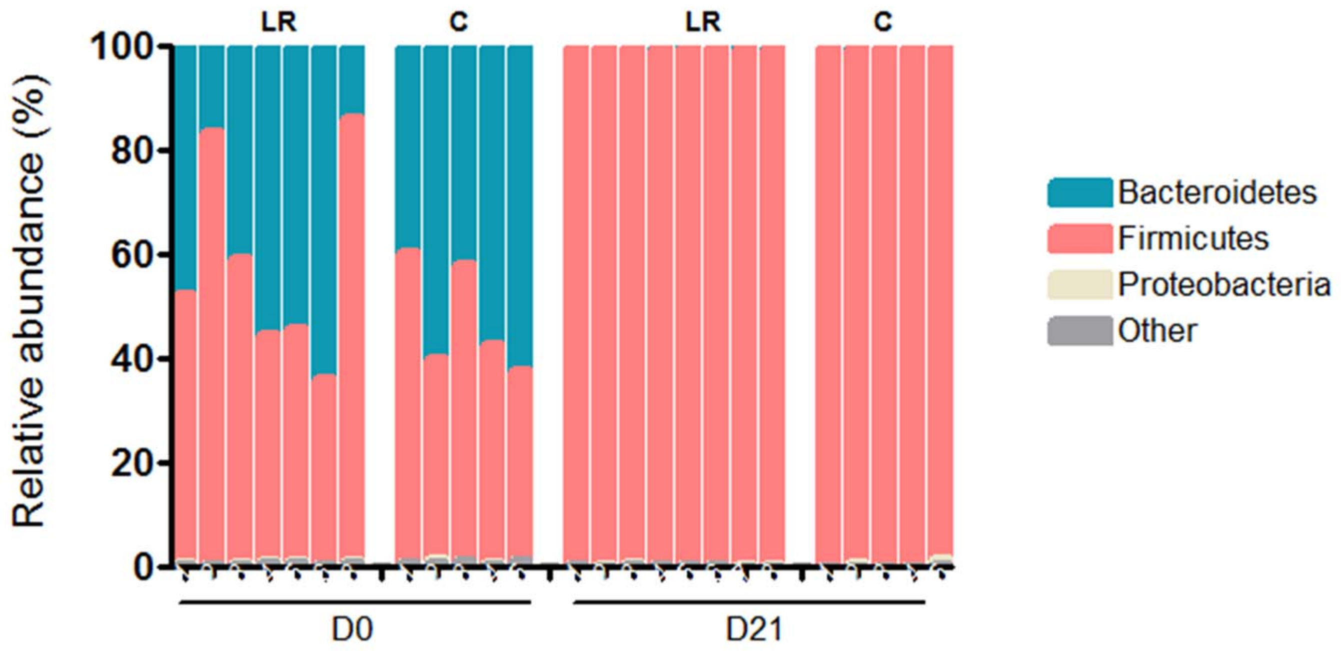

Supplementary Figure S6. Effect of supplementation with *L. rhamnosus* CNCM I-3690 on the microbiota. Relative abundance of the major phyla (>0.01%) at D0 and D21 for control (C) and *L. rhamnosus* (LR) supplemented groups in absence of *E. faecalis* V583. Each bar represents the microbiota of an individual mouse (n = 5 and 8 for the control and *L. rhamnosus* group, respectively).

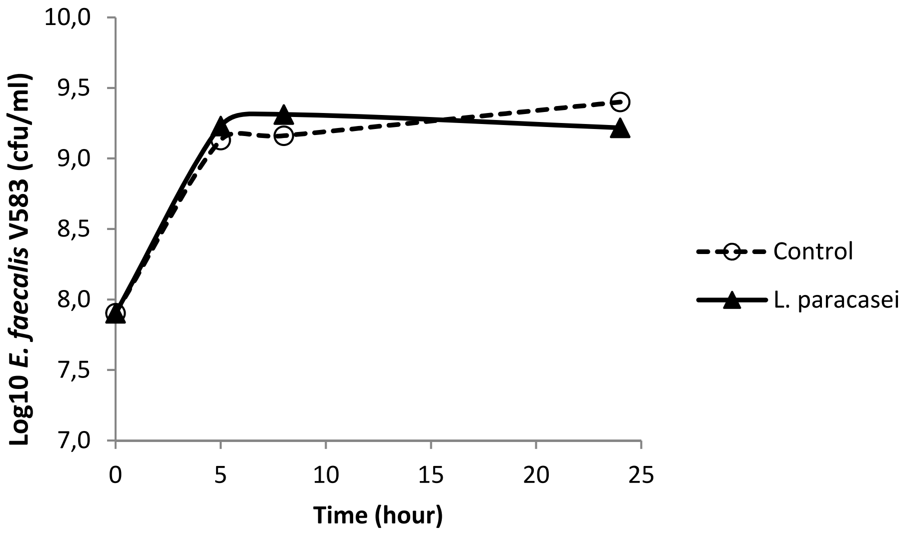

Supplementary Figure S7. *In vitro* study of the impact of *L. paracasei* CNCM-I 3689 supernatant on *E. faecalis* V583 growth. Growth curves of *E. faecalis* V583 in conditioned medium corresponding to M17G supplemented with 50% of MRS (control) or the filtered supernatant of an overnight *L. paracasei* CNCM-I 3689 culture (L. paracasei) monitored by plating at 5, 8 and 24h of growth. A representative experiment of three is shown.

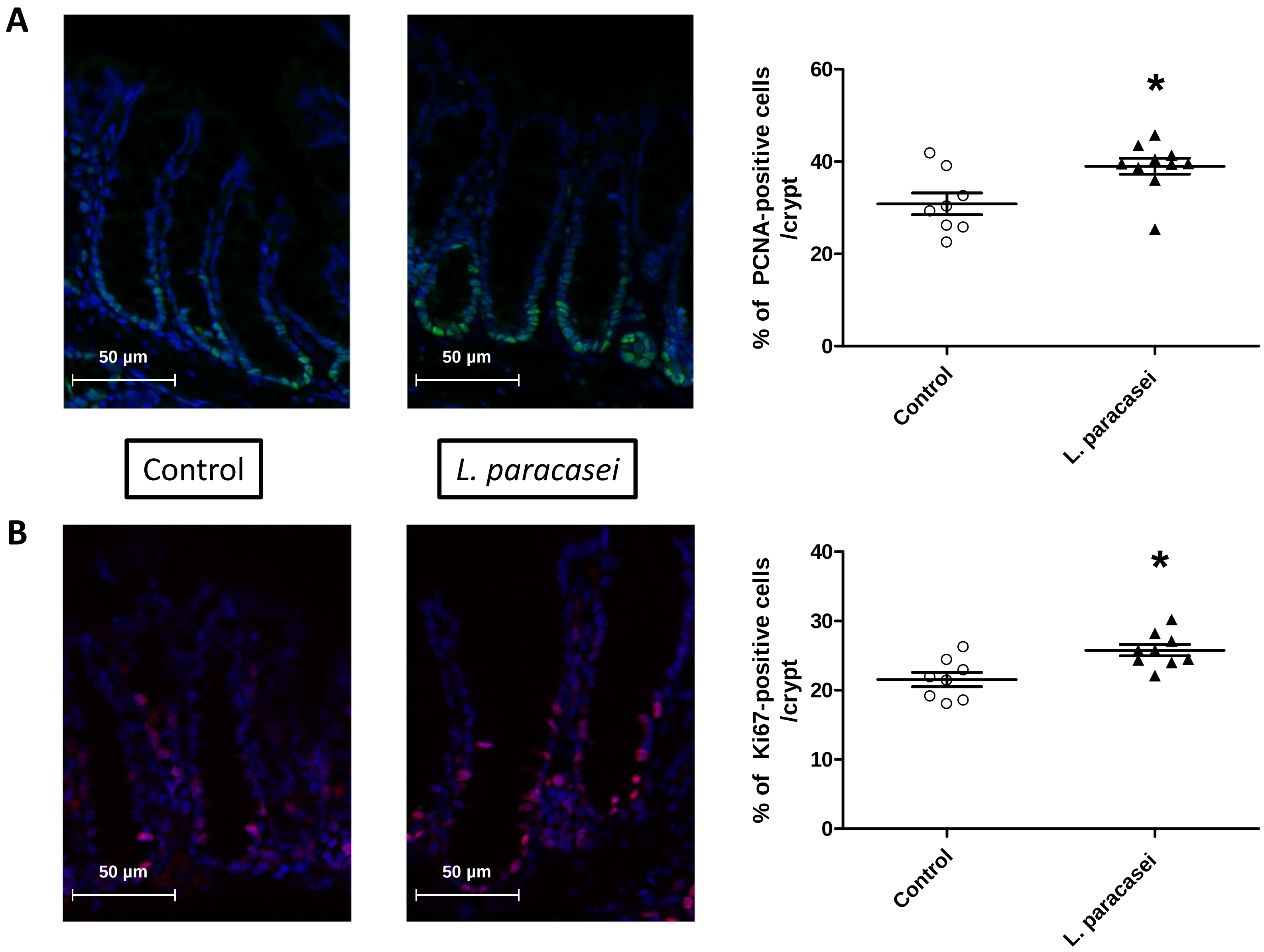

Supplementary Figure S8. *L. paracasei* CNCM I-3689 supplementation impacts on proliferation levels in the colon. (A) Representative immunofluorescence staining for PCNA on the left and percentages of PCNA-positive cells/crypt on colonic sections on the right from control or *L. paracasei* groups of mice (n = 7 to 8) (B) Representative immunofluorescence staining for Ki67 on the left and percentages of Ki67-positive cells/crypt on colonic sections on the right from control or *L. paracasei* groups of mice (n = 7 to 8). *significantly different from control mice values ($P < 0.05$).

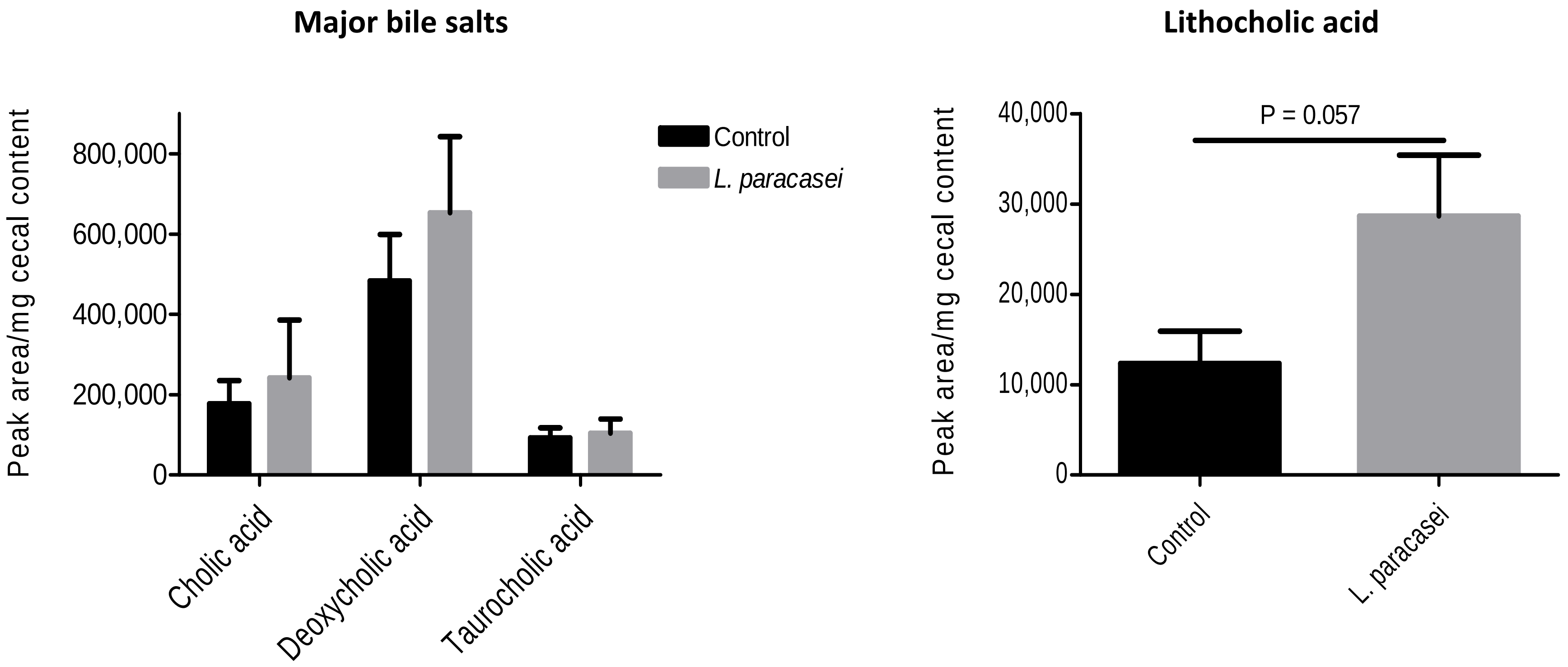

Supplementary Figure S9. Abundance of bile acids detected in cecal samples at D21 in presence of *E. faecalis* V583 (Trial 3). Major bile acids included cholic acid, deoxycholic acid, taurocholic acid, lithocholic acid (n=5-8 mice/group). Statistical comparison between the control and the *L. paracasei* CNCM I-3689-supplemented group was performed using Mann–Whitney test.
